# Larval connectivity patterns of the North Indo-West Pacific coral reefs

**DOI:** 10.1371/journal.pone.0219913

**Published:** 2019-07-23

**Authors:** Patrick R. Pata, Aletta T. Yñiguez

**Affiliations:** Marine Science Institute, University of the Philippines Diliman, Quezon City, Philippines; Swansea University, UNITED KINGDOM

## Abstract

Coral reefs of the North Indo-West Pacific provide important ecosystem services to the region but are subjected to multiple local and global threats. Strengthening management measures necessitate understanding the variability of larval connectivity and bridging global connectivity models to local scales. An individual-based Lagrangian biophysical model was used to simulate connectivity between coral reefs for three organisms with different early life history characteristics: a coral (*Acropora millepora*), a sea urchin (*Tripneustes gratilla*), and a reef fish (*Epinephelus* sp). Connectivity metrics and reef clusters were computed from the settlement probability matrices. Fitted power law functions derived from the dispersal kernels provided relative probabilities of connection given only the distance between reefs, and demonstrated that 95% of the larvae across organisms settled within a third of their maximum settlement distances. The magnitude of the connectivity metric values of reef cells were sensitive to differences both in the type of organism and temporal variability. Seasonal variability of connections was more dominant than interannual variability. However, despite these differences, the moderate to high correlation of metrics between organisms and seasonal matrices suggest that the spatial patterns are relatively similar between reefs. A cluster analysis based on the Bray-Curtis Dissimilarity of sink and source connections synthesized the inherent variability of these multiple large connectivity matrices. Through this, similarities in regional connectivity patterns were determined at various cluster sizes depending on the scale of interest. The validity of the model is supported by 1) the simulated dispersal kernels being within the range of reported parentage analysis estimates; and, 2) the clusters that emerged reflect the dispersal barriers implied by previously published population genetics studies. The tools presented here (dispersal kernels, temporal variability maps and reef clustering) can be used to include regional patterns of connectivity into the spatial management of coral reefs.

## Introduction

The North Indo-West Pacific (NIWP) is an archipelagic region composed of several marginal seas, narrow straits, and shallow bays that host the highest levels of biodiversity and a significant portion of the global coral reef area [1,2]. These coral reefs provide various ecosystem services including reef fisheries, tourism, shoreline protection, and natural products[3,4]. Though heavily tied to the cultural consciousness and the economic development of the region, most coral reefs are highly threatened by overfishing, destructive fishing, siltation, pollution, crown-of-thorns sea star infestation, coral diseases, thermal stress, and ocean acidification [3,5,6], leading to diminishing coral cover by 1–2% per year in the Indo-Pacific [7]. Various management efforts including watershed management, fisheries regulation, reef restoration, marine protected areas, no-take reserves, and integrated coastal management could mitigate current damages and ensure the resilience of coral reef ecosystems in the region [8,9]. However, the global area of protected marine habitats is still far from conservation targets [10,11]. The expansion of management measures to meet conservation goals necessitates that decisions in identifying priority areas for protection should be science-based [12,13]. Larval connectivity is not often included as a criterion [12,14–16] among the multiple ecosystem features utilized to determine which areas should ideally be protected [8,17].

Larval connectivity, henceforth referred to as connectivity, is the exchange of individuals between populations which results from processes involving larval production, transport by currents, larval behaviors, and post-settlement conditions. Population growth, regulation, and recovery from disturbances is dependent on the supply of larvae including both self-recruitment and immigration from other reefs [18–22]. Larval dispersal modeling has been widely used as a practical method to understand connectivity at broad spatial and temporal scales for a large range of organisms [23,24]. Combining the biology and physical environment in models is a powerful tool to accommodate the complex interaction of factors that drives variability in population connectivity [25]. Although modelling cannot provide the certainty of empirical methods, proper model design and parameterization can be sufficient as a best-guess approach to complement information from empirical studies [24] and provide insights into influential factors that should be further investigated.

Connectivity has been found to have high spatiotemporal variability due to a variety of physical and biological factors [18–21,26,27]. Connectivity models which included the NIWP or parts of it have provided insights into the relevant factors of the area, as well as on how its reefs contribute to regional connectivity in the Indo-West Pacific (IWP). Treml et al. [28] highlighted the role of biological characteristics such as reproductive output, reproductive timing and pelagic larval duration in determining dispersal patterns. For Melbourne-Thomas et al. [29], connectivity patterns were broadly similar for reefs in the South China Sea (SCS) side of the Philippines across a range of larval behavior and mortality rates, although the influence of variability due to the spawning period was noted. Seasonal changes and their interaction with the spawning period of the coral *Acropora millepora* was also the source of variability in connectivity patterns for the SCS [30]. The reefs of the Coral Triangle also exhibit relatively high levels of connectivity due to circulation and geographic features [28,31].

The variability in connectivity patterns in the highly diverse and complex area of the NIWP need to be better understood to gain a more detailed picture of between-basin and within-basin larval transport. This would contribute to possible management strategies from the regional NIWP scale to the level of local natural parks and community-based marine protected areas (MPAs). Given that different organisms may produce different connectivity patterns, the management of coral reef ecosystems should consider multiple species towards a metacommunity approach to connectivity [32]. Thus, synthesizing information from a range of organisms would result in a more holistic characterization of connectivity [33,34]. This integration can be derived by analyzing the resulting patterns from biophysical connectivity models.

This study provides a mesoscale analysis of connectivity variability in the NIWP that could be considered in marine resource conservation and management decisions at both regional and more local scales. In particular, we aimed to (1) characterize how connectivity in the NIWP varies between three functionally different coral reef organisms representing a range of early life history conditions, (2) assess the sensitivity of the results to possible seasonal and interannual circulation variabilities, and (3) present a way to identify reef clusters which could serve as management units that integrate the connectivity features based on between-reef similarities in sources and sinks across the three model organisms.

## Methodology

The connectivity model simulating larval dispersal was written in Java, utilizing the MASON simulation toolkit [35]. The model’s overview, design concept, and details [36] are specified in S1 Appendix. Simulations were made for three model organisms spanning a range of potential spawning periods: a branching reef-building coral *Acropora millepora*, a reef-associated keystone herbivore sea urchin *Tripneustes gratilla*, and a predatory coral trout *Epinephelus* sp. These organisms were chosen for their key ecological roles in the reef ecosystem and for their economic value in the NIWP. The temporal span of the study covered three representative years to capture interannual variations of circulation [28] likely due to the El Niño Southern Oscillation (ENSO) cycle [37–40]. Simulation years included 2011 (La Niña), 2013 (normal), and 2015 (El Niño).

### Study domain

**Fig 1:**
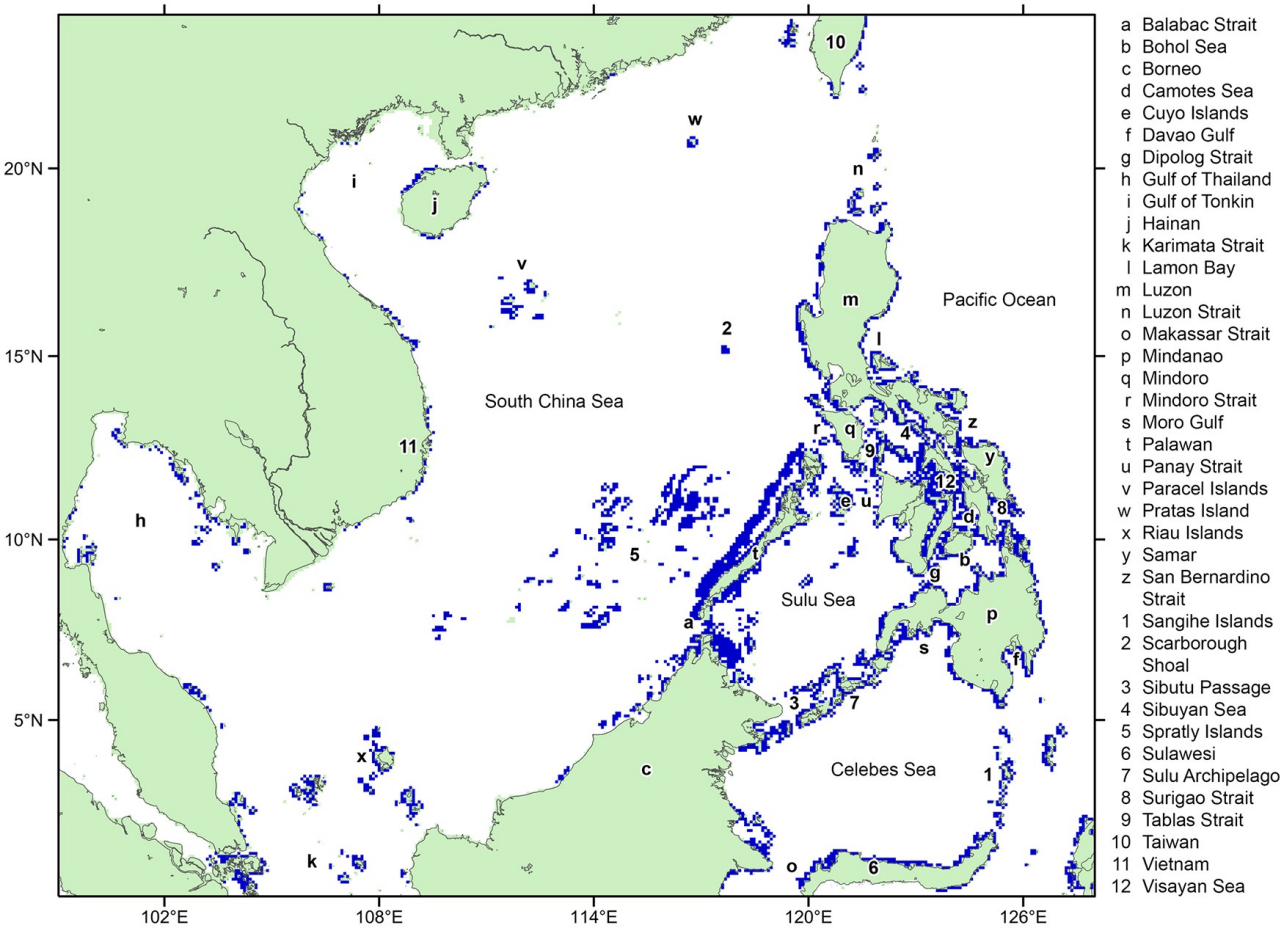
Study domain. Blue cells are rasterized coral reef cells derived from the UNEP World Conservation Monitoring Center (UNEP-WCMC) coral reef database [48]. Green cells are land-masked cells based on the Global HYCOM [49] and gray lines refer to the coast line.

This study covered all coral reef areas (Fig 1) of the NIWP region (0°–24°N, 99°–128° E), including the reefs of Vietnam, Cambodia, Southern China, the Gulf of Thailand, Philippines, Northern Sulawesi, and Northern Malaysia. The modelling domain was set to reduce the loss of potential settling larvae at model boundaries [41]. The southern, western, and northern edges of the study domain were mostly land boundaries outlining the coasts of Southern China and Southeast Asia. Transport of larvae across the water boundaries towards the domain were assumed to be limited given the general direction of water flux across the Taiwan Strait [42,43], Singapore Strait [44], Karimata Strait [45], Makassar Strait, and the Maluku Sea [46]. Through these pathways, the NIWP was modelled to generally act as a source of larvae to other biogeographic regions [31,47]. The eastern open water boundary was the western edge of the Pacific. Transport of modelled larvae from the Philippines eastward was barred by the dominant westward flowing North Equatorial Current (NEC) and the boundary currents which bifurcate from the NEC [37], preventing the IWP from directly transporting particles to the central Pacific [47]. Although importation of larvae from Pacific reefs may occur, it is assumed to be negligible in this study.

### Model inputs

The hydrodynamic input used was the surface circulation of daily global Hybrid Coordinate Ocean Model (HYCOM) [49,50] + Navy Coupled Ocean Data Assimilation (NCODA) Global 1/12° Analysis GLBa0.08 output. The global HYCOM has been assessed in Chassignet et al. [50] based on the regional circulation of the North Atlantic. The global HYCOM 18.2 experiment covering circulation from 2003 to 2010 of the Sulu Sea and the Philippine internal seas has been validated in Hurlburt et al. [38]. The GLBa0.08 dataset used has a Mercator-curvilinear horizontal grid format with a resolution of around 0.08° and this was the basis for the gridding of the individual-based model. Land masking was also based on the hydrodynamic data in which cells with numerical values were ocean and land, if otherwise. The resolution was unable to capture some straits because of poor land masking (e.g., Tañon Strait, 10.4°N 123.5°E; Guimaras Strait, 10.8°N 122.8°E) and thus the reefs for these areas were not included in the analysis.

Coral reef locations were identified based on the UNEP World Conservation Monitoring Center (UNEP-WCMC) coral reef database [48]. Individual reef vectors were merged and rasterized into reef cells using QGIS 2.8.1 to fit the model grid. Some coastal reefs, and reefs within small embayments and straits were masked as land cells because of the limited resolution of the hydrodynamic model. Wherever this was the case, the adjacent non-reef cell was converted to a reef cell to at least estimate connectivity of that general area. This resulted in 3,776 reef cells. Reproductive output likely varies significantly between reef cells and spawning periods, but the absolute values for the area are not known. This study assumed that all reef cells have equal degrees of larval productivity which was necessary to compute the relative probability of settlement.

Various biological factors affect larval dispersal, but the most influential ones considered in this study (Table 1) were mortality rate, pelagic larval duration (PLD) [41], pre-competency period [25], and swimming capability [41,51,52]. These were parameterized based on the available information gathered from the NIWP. The broadcast spawning coral *A*. *millepora* was regarded as a short-distance spawner. Both *T*. *gratilla* and *Epinephelus* sp. were considered long-distance spawners and were simulated to produce passively dispersing and actively swimming larvae, respectively. *Acropora millepora* was observed to spawn during the summer months in the Philippines [53] while *T*. *gratilla* spawns year-round [54]. *Epinephelus* sp. may also spawn during different periods of the year, but has peak spawning during the summer months [55]. This study analyzed the results according to four seasons based on the monsoons: northeast monsoon for December, January, and February (DJF), summer for March, April, and May (MAM), southwest monsoon for June, July, and August (JJA), and the transition months of September, October, and November (SON).

**Table 1:** Early life history characteristics of model organisms. Empirical values were based on the representative organisms.

Model Organism	Age of settlement competency (days)	Maximum PLD (days)	Mortality rate (day^-1^) *	Swimming behavior
*Acropora millepora*	3 [56]	60 [56]	0.023	No
*Tripneustes gratilla*	29 [54]	57 [57]	0.024	No
*Epinephelus* sp.	36 [58]	47 [58]	0.029	Yes**

* derived from a half-life equation [44,56]

** sustained swimming speed was computed as 50% of the critical swimming speed which is a function of age

### Individual-based model

Circulation data and reef GIS data were assimilated into a Lagrangian particle-tracking model at a spatial resolution of 0.08° by 0.08° with a model time step interval set to 2,700 seconds based on Kough et al. [59] which used a similar model resolution. This also met the Courant-Friedrich-Lewy condition [41] while not debilitating computation time [18]. Larvae were simulated as particles transported across the model space until the event of settlement. The model was initiated at a high frequency of every five days and daily matrices were averaged per season. One hundred, 250 and 450 larvae were simulated and positioned randomly in each cell for *A*. *millepora*, *T*. *gratilla*, and *Epinephelus* sp., respectively. Based on a calibration exercise (S2 Appendix), these values for spawning dates and number of modelled larvae would not significantly vary the resulting connectivity matrix [26]. These were also similar to Holstein et al. [33] with a comparable set of PLD parameters.

Once spawned, larvae were continually subjected to transport by advection, diffusion, and swimming during each model time step. Advection was based on the current vector at the particle’s location interpolated in space and time. The advection of larvae used a Runge-Kutta 4^th^ order differential equation scheme which modelled particle transport more realistically, especially near land boundaries [41]. Diffusion was a random-walk equation that contributes a transport vector orders of magnitude lower than the advection value. This accounted for sub-grid scale processes and dispersions due to turbulence [60]. A horizontal swimming behavior was applied to *Epinephelus* sp. larvae once reaching the flexion age of 20 days [61]. Each post-flexion *Epinephelus* sp. larvae searched the adjacent grid cells relative to its current position. If reef cells were detected, distances towards the adjacent reef cells were computed and swimming was directed towards the nearest reef cell. The sustained swimming speed was computed as 50% of the critical swimming speed [62]. The latter was derived from an age–swimming speed function [63]. If no reef cell was detected, the swimming module was disabled for the current time step. Once inside a reef cell, swimming was also disabled. This method in modelling larval swimming behavior was based on Wolanski and Kingsford [64]. The inclusion of swimming behavior mediates the dispersal of *Epinephelus* sp., which has been suggested to increase local retention [65,66].

Although passive and active vertical transport are certainly relevant in the dispersal of larvae [65,67], this study only utilized the surface circulation in part for the purpose of simplifying model computation and because of the lack of necessary biological information to parameterize this process. Since excluding vertical migration probably leads to an overestimation of modelled dispersal distance [68], the model output provides an upper limit on the range of potential connectivity patterns [69]. When a larva encountered a land boundary, it was modelled to return to the ocean cell to remain within the model. Upon leaving the model boundaries, the larva was removed from the simulation.

Larval mortality was computed at the end of each model day by randomly drawing a value from 0 to 1 for each larva. If this was below or equal to the mortality rate of the organism (Table 1), the larva was considered dead and removed from the simulation. Upon reaching the maximum PLD and if it is not on a suitable habitat, the larva was also considered dead and removed from the simulation. Once the larva reached the age of settlement competency (Table 1) and was located on a reef cell, settlement could have occurred at a 50% probability similar to Dorman et al. [30]. This attempted to simulate competent larvae passing by a reef cell and not settling on it. Resulting matrices, however, were not sensitive to the value of the settlement probability (Figure B8 in S2 Appendix). A reef cell where each larva settled on was considered as the sink cell. The model continued to run until the PLD value. Past this, all larvae which have not yet settled were assumed to have died. The output file recorded the source reef IDs, sink reef IDs, and the number of larvae simulated to form each connection.

### Metrics of connectivity patterns

The model outputs were recorded as raw connectivity matrices of the total number of simulated larvae that settled from a source site (*i*) to a sink site (*j*) for each simulation. The rows of the matrix constituted the sources while the columns constituted the sinks. The raw connectivity matrix was then converted into a settlement probability matrix according to:

$$P_{set(i,j)}=\frac{N_{i\to j}}{N_{i}},$$ (1)

in which *P*_*set*(*i*,*j*)_ was the settlement probability representing the proportion of larvae exported from a source *i* to a sink *j* relative to the number of larvae released from the source. A total of 36 matrices were generated in this study based on 3 organisms, 4 seasons, and 3 years.

The dispersal kernels for each organism were determined through curve-fitting in MATLAB [70] to provide estimates of the settlement probability given only the distance between reefs. The mean settlement probability of all connections of each source-to-sink 1 km distance bins were used as inputs to explore different curve-fitting functions. For the dispersal kernel computation and to compare the metrics between organisms, the summer matrices were used for *A*. *millepora* while all four seasonal matrices were used for *T*. *gratilla* and *Epinephelus* sp. to reflect the range of known spawning periods of these organisms.

Seven connectivity metrics were used to analyze connectivity patterns: local retention, export and import probabilities, out- and in-degrees, and the average export and import distances. The diagonal of the matrix, *P*_*set*(*i*,*i*)_, was a measure of local retention [21] or the proportion of larvae that was retained or has returned to its natal reef, relative to how many larvae were released from the source. The horizontal sum of the matrix minus local retention was the total export probability of each source, representing the proportion of larvae that settled to other reefs relative to how many larvae were released from the source. The vertical sum of the matrix minus local retention was the estimate of import probability of the sink, representing the likelihood that a reef would receive larvae from other reefs. We also computed the number of unique external connections formed by each reef as either a source or a sink. The out-degree is the number of unique sinks of each source while the in-degree is the number of unique sources of each sink. The mean distances of exports or imports of each reef were the weighted averages of all source-to-sink or sink-to-source Euclidian distances, similar to Wren et al. [71], in which the weights were based on the settlement probabilities.

The mean direction of exports was also computed for temporal analysis. This was based on the mean Euclidean source-to-sink directions weighted by the settlement probabilities. For this, only long-distance connections beyond 13 km (i.e., more than 1 grid cell away) were considered in the averaging to remove local retention and adjacent-cell connections.

### Comparison of metrics

The Kruskal-Wallis one-way analysis of variance and the post-hoc Dunn’s test were used to compare the distributions of connectivity metrics testing for significant differences in the distribution between model organisms and between seasonal matrices. Pearson correlation coefficient was used to explore the relationships of connectivity metrics between model organisms and between seasonal matrices.

Circular analysis was employed to compare the distributions of the mean direction of exports of each reef cell between seasons and years for each organism. A multi-sample test for equal median directions, a circular analogue of the Kruskal-Wallis test [72], was used to compare temporal directionality between distributions. The temporal variability of the mean direction of exports of each reef cell was computed separately for seasonal and interannual variability. For each organism and year, the standard deviation was computed across seasons and then averaged (N = 9) to derive the seasonal variability. Likewise, it was computed for each organism and season and then averaged (N = 12) to derive the interannual variability. All statistical analyses were done using MATLAB.

### Synthesizing spatiotemporal connectivity patterns

The settlement probability matrices were subjected to agglomerative cluster analysis to explore possible clusters of reefs which covary in connectivity patterns based on similarities of the sources and sinks of each reef cell. The distance metric of the cluster analysis was based on the Bray-Curtis dissimilarity (BCD) equation [73] in which each source and sink connection of a reef cell across a set of matrices was considered a “species.” Thus, the BCD between cells *i* and *j* was:

$${BCD}_{ij}=1-\frac{2C_{ij}}{S_{i}+S_{j}},$$ (2)

wherein *C*_*ij*_ was the sum of the smaller magnitudes of connections shared between both cells, and *S* was the sum of all connections in each cell. A *BCD*_*ij*_ value close to zero meant that reef cells had similar connections and variability across the matrices used in the analysis while a value close to one meant that cells had dissimilar connections. The BCD matrix used here was based on the known spawning periods of each organism (summer matrix for *A*. *millepora* and the annually-averaged matrix for *T*. *gratilla* and *Epinephelus* sp.) to reflect how managing coral reef ecosystems could cut across multiple key organisms.

The resulting BCD matrix was subjected to a dynamic tree cut algorithm implemented in the R programming language [74] using default parameter values and different minimum cluster sizes to illustrate possible scales of management. The minimum cluster size (MCS) parameter represents how similar reefs should be to form a cluster [74]; lower values require more similarity. The dynamic tree cut algorithm was used since it can handle complex dendrograms with possible nested clusters and it allowed for very high dissimilarity branches to be unclustered.

## Results

### Between-organism differences in dispersal kernels and connectivity metrics

**Fig 2:**
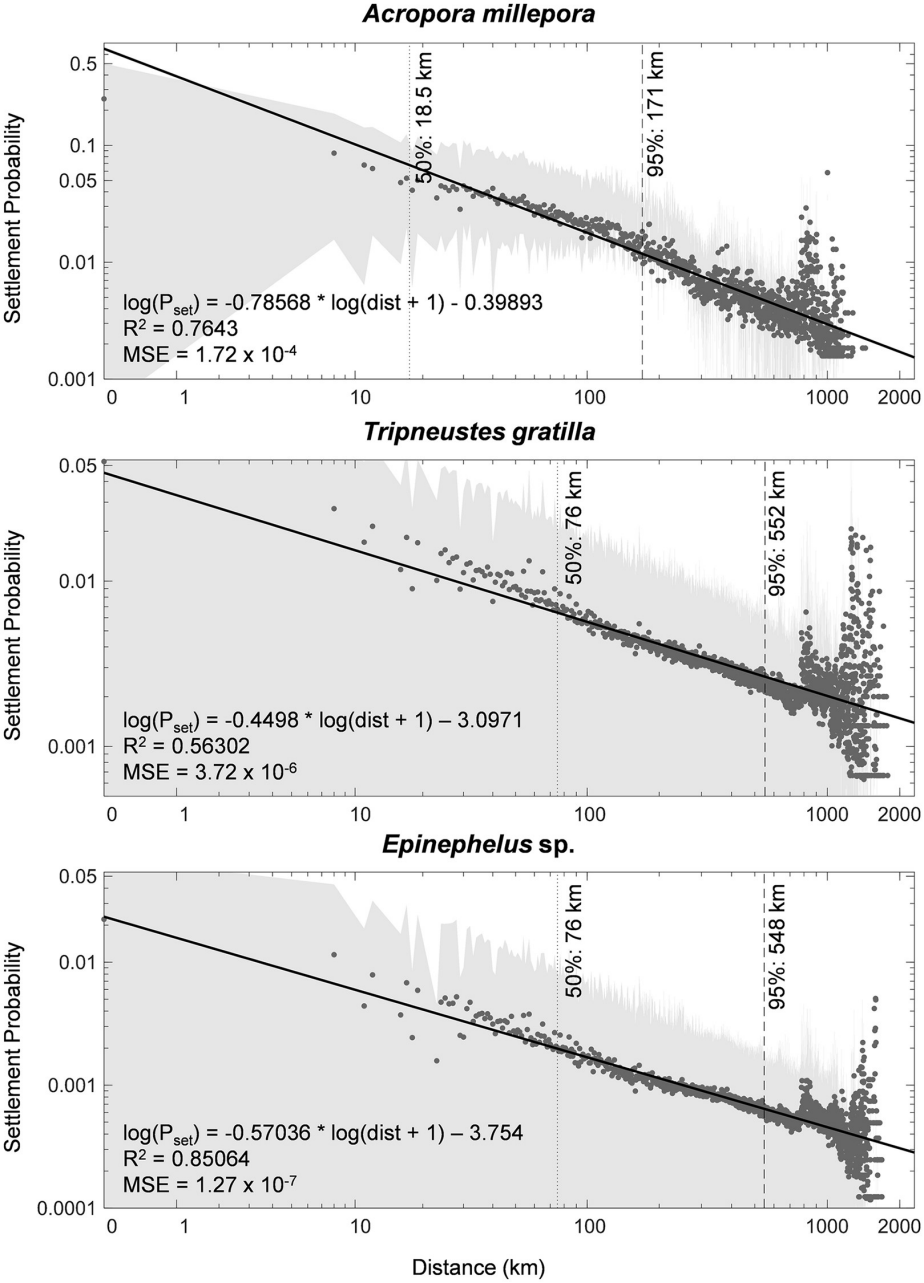
Fitted power law functions from dispersal kernels of each model organism. Dark gray dots are the mean settlement probability (*P*_set_) of each source-to-sink distance (dist) with gray areas showing the standard deviation. Note that the axes are in log scale and the limits of the y-axis varies. Verticals lines show the distance of connections at the median (dotted) and at 95^th^ percentile (dashed) of connections.

Fitted power law functions for the dispersal kernels provided the highest coefficient of determination and the lowest mean standard error (Fig 2). These dispersal kernels demonstrated that closer connections had higher probabilities which then tapered off with distance. For all model organisms, 95% of larvae settled to a third of the maximum settlement distance. Half of the *A*. *millepora* larvae settled within 18.5 km or around two model cells away and most settled less than 171 km from their natal reefs. *Tripneustes gratilla* and *Epinephelus* sp. had similar settlement distances despite the latter having a longer larval duration. Relative to the median, the settlement probability at the 95^th^ percentile was more than half and the probabilities at the farthest settlement distances were an order of magnitude lower for all organisms.

The best fit of power law equations was the mid-range from the median to the 95^th^ percentile; beyond which, there were spikes of higher export probabilities at distances of around 800 km and 1,300 km (Fig 2). The 800-km connections mostly represented cross-basin transport around the Celebes Sea, connections of offshore reefs around the South China Sea, and direct links from the Surigao Strait to eastern Borneo and from Samar and northeastern Luzon to Taiwan. The 1,300-km connections were larval transport between reefs of southern Vietnam with reefs at Mindoro Island and northwestern Luzon and direct transports from eastern Samar to the Makassar Strait.

**Fig 3:**
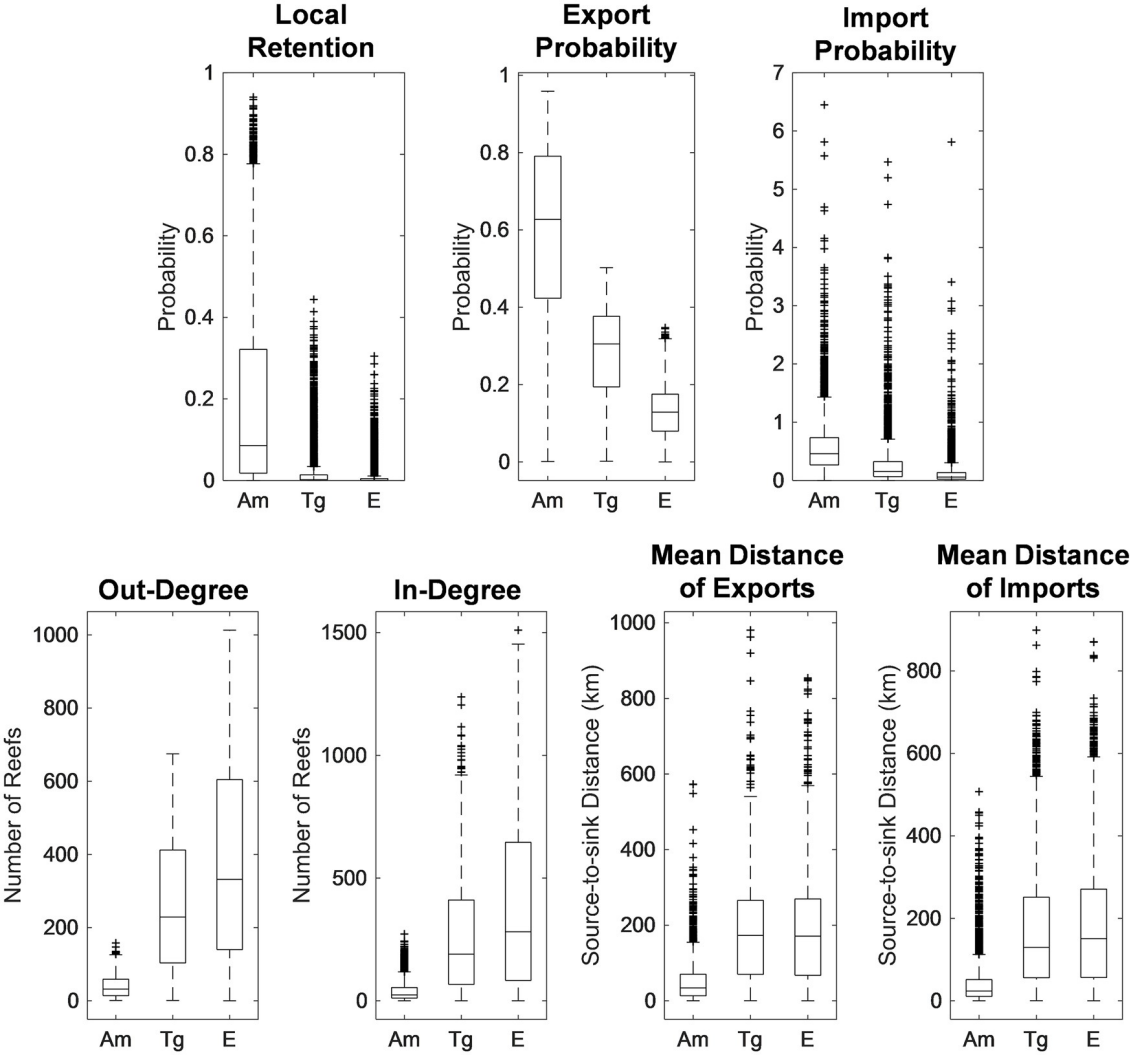
Comparison of connectivity metric distributions between organisms. All distributions were significantly different (p < 0.001). Am: *Acropora millepora*, Tg: *Tripneustes gratilla*, E: *Epinephelus* sp. Boxes display the first and third quartile spread of the data, the central line indicates the median, and the whiskers and outliers denote the range of values.

Comparing the distributions of the connectivity metrics of each reef cell (N = 3,776) using Kruskal-Wallis one-way analysis of variance showed that distributions of all metrics (Fig 3) were significantly different (p-values < 0.001) between organisms. Post-hoc Dunn’s test showed that all pairs were also significantly different (Table A in S3 Appendix) except for mean distance of export and imports between *T*. *gratilla* and *Epinephelus* sp. The values for local retention, export probability and import probability decreased with increasing age of settlement competency (*A*. *millepora* > *T*. *gratilla* > *Epinephelus* sp). On the contrary, *A*. *millepora* scored lowest in terms of the range of external connections for out-degree, in-degree, mean distance of export, and mean distance of imports. *Tripneustes gratilla* and *Epinephelus* sp. performed similarly for these metrics. Most of the between-organism pairs for all metrics were highly correlated (Table 2) except for the moderately correlated (r < 0.50) *A*. *millepora* versus *Epinephelus* sp. value for import probability. The values for *T*. *gratilla* and *Epinephelus* sp. were more correlated with each other compared with *A*. *millepora*.

**Table 2:** Pearson correlation coefficients of the connectivity metric of each reef cell between model organisms. All correlations were significant (p-values < 0.001).

Connectivity Metric	*Acropora millepora* vs *Tripneustes gratilla*	*Acropora millepora* vs *Epinephelus* sp.	*Tripneustes gratilla* vs *Epinephelus* sp.
Local Retention	0.796	0.723	0.961
Export Probability	0.554	0.566	0.886
Import Probability	0.520	0.458	0.883
Out-degree	0.752	0.739	0.985
In-degree	0.838	0.748	0.942
Mean distance exports	0.711	0.695	0.940
Mean distance imports	0.690	0.646	0.9242

### Temporal variability in connectivity patterns

The seasonal matrices for each organism were significantly different for all metrics (p-values < 0.001) (Figure A in S4 Appendix) although not all between-season pairs were significantly different (Table B in S3 Appendix). Often, the SON and DJF matrices were similar for some metrics and organisms and the MAM and JJA matrices had similar metric distributions for *A*. *millepora*. Comparing the annual and seasonal connectivity matrices, the metrics derived from the annually-averaged matrices of each model organism were highly correlated with the metrics from the seasonal matrices (Table 3). The southwest monsoon (JJA) matrices were most dissimilar with the annual average, but still had a high correlation coefficient. The summer matrices (MAM) were most correlated with the annual average. The values for each of the different connectivity metrics were generally correlated between seasonal matrices for all model organisms but with some exceptions. Both *T*. *gratilla* and *Epinephelus* sp. had relatively lower correlation coefficient values between seasons compared to those of *A*. *millepora*. The most salient seasonal differences were import probability and in-degree of both *T*. *gratilla* and *Epinephelus* sp. which were only weakly to moderately correlated between JJA vs DJF and JJA vs SON.

**Table 3:** Pearson correlation coefficients of the connectivity metric values of each reef cell between different temporal matrices for each model organism. All correlations were significant (p-values < 0.001).

	Local Retention	Export Probability	Import Probability	Out-Degree	In-Degree	Mean Distance Exports	Mean Distance Imports
*Acropora millepora*							
DJF vs. MAM	0.921	0.876	0.671	0.857	0.829	0.704	0.749
DJF vs. JJA	0.800	0.685	0.311	0.664	0.593	0.437	0.606
DJF vs. SON	0.949	0.921	0.814	0.918	0.928	0.812	0.900
MAM vs. JJA	0.894	0.841	0.610	0.792	0.742	0.724	0.720
MAM vs. SON	0.939	0.892	0.715	0.861	0.794	0.704	0.726
JJA vs. SON	0.874	0.782	0.526	0.740	0.678	0.575	0.646
Annual vs. DJF	0.956	0.932	0.836	0.905	0.920	0.848	0.917
Annual vs. MAM	0.978	0.964	0.883	0.938	0.924	0.871	0.869
Annual vs. JJA	0.928	0.883	0.729	0.866	0.804	0.771	0.807
Annual vs. SON	0.979	0.961	0.912	0.929	0.917	0.898	0.926
*Tripneustes gratilla*							
DJF vs. MAM	0.807	0.800	0.627	0.864	0.706	0.702	0.732
DJF vs. JJA	0.455	0.440	0.093	0.670	0.335	0.424	0.610
DJF vs. SON	0.911	0.918	0.455	0.440	0.093	0.876	0.864
MAM vs. JJA	0.758	0.767	0.490	0.829	0.635	0.726	0.713
MAM vs. SON	0.846	0.840	0.650	0.832	0.665	0.753	0.710
JJA vs. SON	0.568	0.536	0.198	0.640	0.400	0.545	0.576
Annual vs. DJF	0.892	0.893	0.830	0.880	0.810	0.830	0.883
Annual vs. MAM	0.957	0.957	0.867	0.961	0.888	0.902	0.868
Annual vs. JJA	0.779	0.771	0.572	0.894	0.747	0.780	0.790
Annual vs. SON	0.934	0.930	0.868	0.855	0.804	0.891	0.869
*Epinephelus sp*.							
DJF vs. MAM	0.747	0.706	0.561	0.815	0.643	0.67	0.661
DJF vs. JJA	0.373	0.288	0.08	0.636	0.324	0.433	0.606
DJF vs. SON	0.76	0.773	0.675	0.856	0.814	0.77	0.756
MAM vs. JJA	0.739	0.723	0.501	0.864	0.693	0.766	0.723
MAM vs. SON	0.875	0.864	0.719	0.918	0.731	0.813	0.736
JJA vs. SON	0.707	0.649	0.419	0.861	0.615	0.723	0.699
Annual vs. DJF	0.824	0.795	0.759	0.864	0.784	0.797	0.855
Annual vs. MAM	0.953	0.947	0.866	0.942	0.862	0.903	0.845
Annual vs. JJA	0.799	0.779	0.635	0.905	0.78	0.83	0.807
Annual vs. SON	0.946	0.944	0.884	0.959	0.888	0.929	0.887

Temporal differences of the distributions of the mean direction of exports at each reef for each organism were found to be significantly different between seasons and between years (Figures B-D in S4 Appendix). During the transition months until the northeast monsoon (DJF), connections were mostly westward with eastward settlement limited to a few source reef cells. This is reversed during the southwest monsoon when connections were dominantly eastward. During the summer months, the connections were more spread out to other directions, but the highest distributions were still westward. Between years, seasonal connections maintained their general directional pattern with the mean direction vector of each distribution shifted by a few degrees.

**Fig 4:**
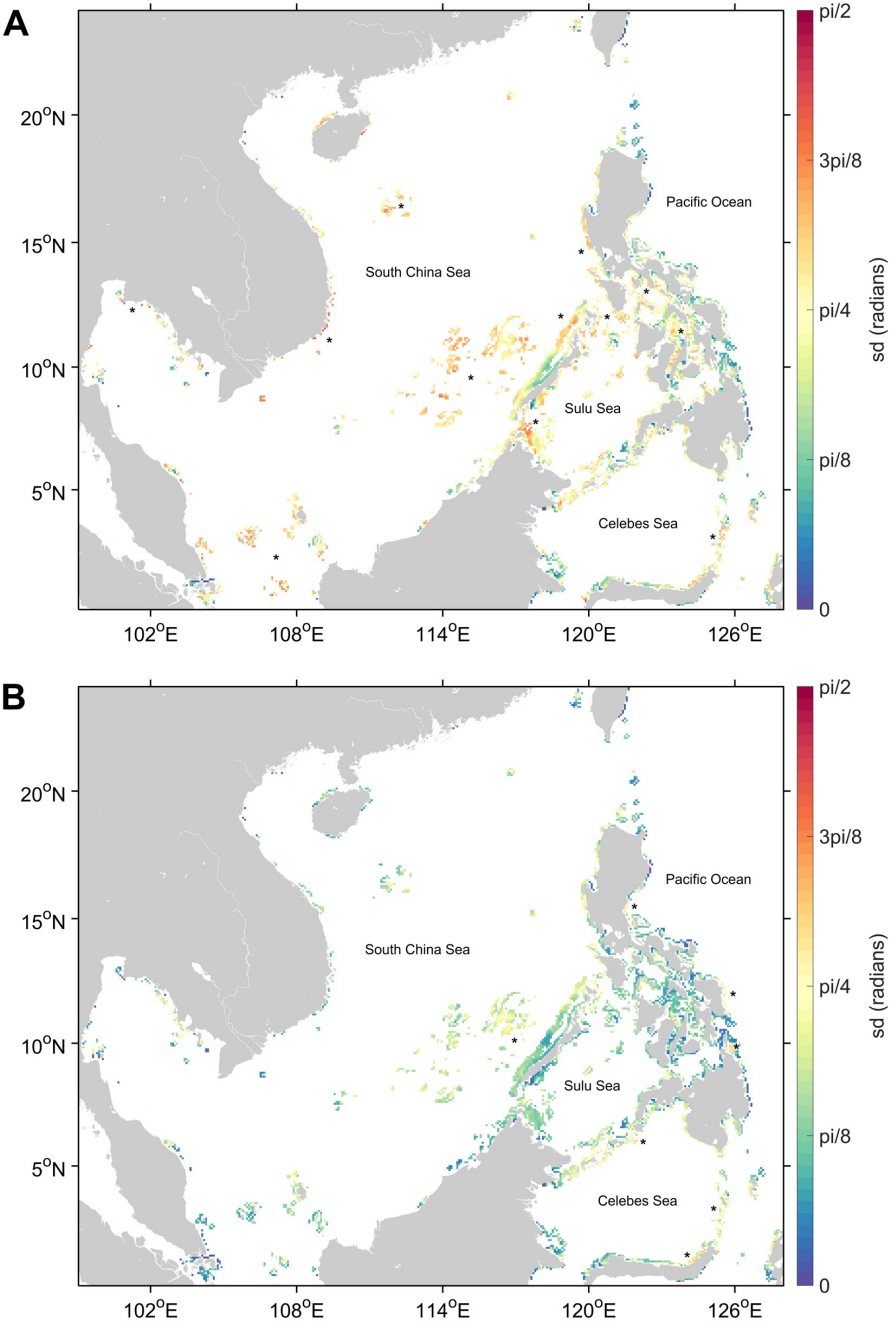
Variability of mean direction of exports at each reef cell. (A) shows the average standard deviation between seasons, (B) shows the average standard deviation between years. Areas marked with * indicate reefs of high variability mentioned in the text.

Seasonal variability (Fig 4A) was generally higher at areas of distinct current reversals including the Karimata Strait, offshore reefs around the South China Sea, the Vietnamese coast, southwestern Luzon, Sangihe Islands, and most reefs around the Sulu Sea. Seasonal variability was also high for more restricted areas like the internal seas of the Philippines and the Gulf of Thailand. Connection directions were more consistent across seasons for reefs at the eastern Luzon and eastern Mindanao coasts. Interannual variabilities (Fig 4B) were lower than seasonal variabilities. Areas which had relatively higher interannual variability were eastern Samar, northwestern Lamon Bay (14.5°N 121.8°E), Dinagat Sound (9.8°N 125.8°E), northern Celebes Sea, Sangihe Islands and northeastern Sulawesi, and the central Spratly Islands (10.9°N 116.8°E).

### Clusters of reefs with covarying connectivity patterns

The dendrogram based on connectivity could be cut at different levels through the MCS parameter of the dynamic tree cut algorithm depending on the possible spatial scale of interest or management. As the number of clusters increased resulting from decreasing MCS values, the range in cluster sizes (number of reef cells per cluster) decreased and fewer cells were excluded from clustering (Table 4). The cluster analysis organically resulted in clusters characterized by geographic location and circulation features even if these were not included as part of the clustering criteria. The largest cluster which emerged at MCS = 160 was a singular cluster (Figure A in S5 Appendix) composed of the reefs of Vietnam, the Paracel Islands, Spratly Islands, northwestern Borneo, the west Philippine Sea, and Sulu Sea. These reefs were most similar regionally in terms of having high interconnectivity, are general larval sources to the southern South China Sea and the Celebes Sea, and serve as larval sinks for the eastern and internal seas of the Philippines.

**Table 4:** Clustering details at various minimum cluster size (MCS) parameter values. The size of clusters was measured by the number of reef cells included in the cluster.

MCS parameter	Number of Clusters	Minimum Cluster Size	Maximum Cluster Size	Mean Cluster Size	SD of Cluster Sizes	Number of Unclustered Cells
1	924	2	19	4	2.3	11
5	385	5	33	9.7	4	29
10	194	10	48	19.1	7.2	64
15	134	15	67	27.7	9.8	64
20	101	20	128	36.6	16	80
25	70	26	128	51.2	22.6	192
50	34	51	270	100.5	43.6	359
75	19	80	369	178.7	80.3	381
100	13	104	471	248.6	122.5	544
125	7	178	877	420.1	255.9	835
150	5	178	877	459.8	290.1	1477
160	1	1454	1454	1454	0	2322

**Fig 5:**
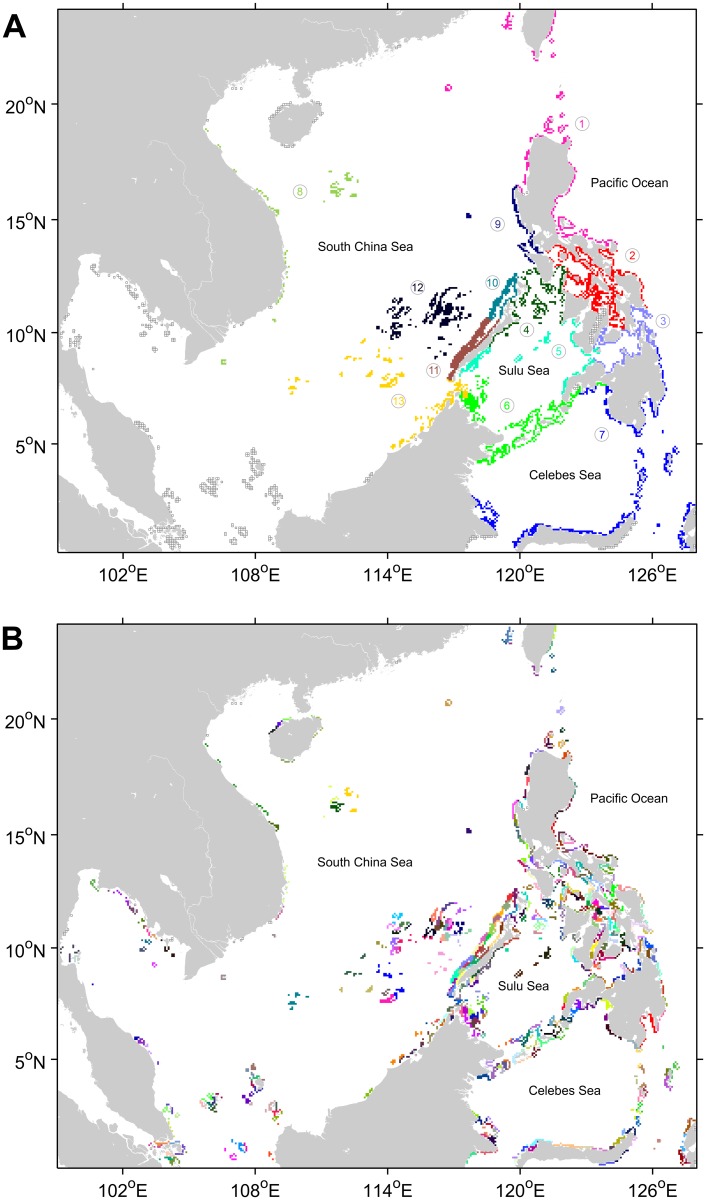
Clusters of covarying connectivity patterns based on the Bray-Curtis Dissimilarity matrix of the source and sink connections of the three model organisms. The dendrogram was cut at (A) MCS = 100 and (B) MCS = 5. Each color represents a unique cluster. Reefs boxed in gray were outliers excluded from the clustering. Clusters identified in (A) are (1) Northern Luzon and Taiwan, (2) Philippine internal seas and San Bernardino Strait, (3) Bohol Sea and Surigao Strait, (4) Northern Sulu Sea, (5) Central Sulu Sea, (6) Southern Sulu Sea, (7) Celebes Sea, (8) Vietnam and Paracel Islands, (9) Western Luzon, (10) Northwestern Palawan, (11) Southwestern Palawan, (12) Northern Spratly Islands, and (13) Southern Spratly Islands and Western Borneo.

A total of 13 clusters emerged at MCS = 100 (Fig 5A). The Northern Luzon and Taiwan formed one cluster (cluster 1). This cluster was mainly a source of larvae to the northern South China Sea as the Kuroshio intrudes through the Luzon Strait. The Philippine internal seas and San Bernardino Strait formed a cluster of reefs (cluster 2) which were highly interconnected but had relatively limited external connections. The Bohol Sea and Surigao Strait formed a cluster (cluster 3) differentiating it as the main sink of larvae from the Pacific Ocean and a source of larvae of the Sulu Sea. The Sulu Sea was subdivided into three clusters representing the northern (4), central (5), and southern (6) portions. The northern cluster formed connections mostly with the northern South China Sea through the Mindoro Strait. The central cluster was the primary sink of the Bohol Sea through the Dipolog Strait [75]. The southern cluster composed of the Sulu Archipelago and northeastern Borneo were highly interconnected with the southern South China Sea through the Balabac Strait and with the Celebes Sea. The Celebes Sea cluster (cluster 7) consistent of reefs which were most influenced by the Mindanao current and its associated eddies.

The South China Sea was subdivided into six clusters which differentiated near-coast reefs from offshore reefs. The cluster composed of Vietnam and Paracel Islands (cluster 8) highlighted the reefs which were strong larval sources during the southwest monsoon and conversely, the main larval sinks of the northern South China Sea and northeastern Luzon during the northeast monsoon. The Western Luzon cluster (9) similarly was the sink of the Vietnam-Paracel Island cluster during the southwest monsoon and the source of larvae to the South China Sea during the rest of the year. The western Palawan shelf was subdivided into the northern reefs (cluster 10), connecting mostly with the northern South China Sea and the Mindoro Strait, and the southern reefs (cluster 11), connecting with the southern South China Sea and the Balabac strait. The Spratly Islands were also subdivided to the northern cluster (12) connecting with the northern South China Sea and the southern cluster (13) including Western Borneo mostly connected with the southern South China Sea and the Celebes Sea.

The Karimata Strait cluster emerged at MCS = 75 (Figure B in S5 Appendix) which have relatively isolated reefs connecting mostly with the Spratly Islands and the Gulf of Thailand as well. The Celebes Sea was divided into a generally northern-side and a Sulawesi-side at the same MCS. The southern Sulu Sea cluster was divided into the sides of the Sibutu Passage at MCS = 50 (Figure C in S5 Appendix). The Philippine internal seas were similarly divided into the North and South Sibuyan Sea, Visayan Sea, and Camotes Sea while Bohol Sea was also bisected into the northern and southern sides of the Bohol Jet. Most of the individual bays and islands were separated as their own clusters at MCS = 20 (Figure D in S5 Appendix). The Gulf of Thailand and Hainan reefs, which had limited external connections with the South China Sea also emerged as clusters. At MCS = 5, 385 clusters were formed (Fig 5B) with an average size of 9.7 ± 4 reef cells. This decreased the scale to about 10% of the original matrix while retaining most of the simulated regional connectivity patterns.

## Discussion

The North Indo-West Pacific region is characterized by complex circulation and distinct monsoonal systems. These conditions together with differences in early life history-characteristics gave rise to variations in patterns of connectivity. Interestingly though, these patterns were generally correlated with each other, pointing to the large influence of the spatial configuration of reefs on connectivity in the NIWP. This increases support for the integration of the different matrices and coming up with clusters representing covarying connectivity patterns.

### Influence of early life history characteristics on connectivity

The dispersal kernels provided a useful overview of relative settlement probabilities between reefs of the NIWP given only their distance, and highlight some variabilities due to early life history traits. These showed that the earlier onset of settlement for *A*. *millepora* when offshore dispersal was still limited, led to higher local retention, echoing the results of other dispersal modelling studies [25,47]. Long-distance connections, though rare, were also observed for the *A*. *millepora* simulations. This is a likely scenario given that the lipid contents of some coral eggs may extend PLDs to 100 days [76,77]. The relatively higher probability long-distance connections around 800 km and 1,300 km made by less than 5% of the settled larvae were most likely the result of offshore transport and not readily encountering reefs when larvae were already competent to settle. The high probability of settling close to the natal reef while still forming connections at more than 1,000 km suggest that developing local strategies in protecting the linkages of larval supply may also benefit downstream reefs at a regional scale [31,47]. Conversely, more unique connections at farther distances were recorded for *T*. *gratilla* and *Epinephelus* sp. when the age of settlement competency was delayed by a month. The sensitivity analysis (S2 Appendix) showed that the model was more sensitive to settlement age, even by one-day variation, compared to the PLD. This was expected since transport of larvae in a single day may greatly reshape the dispersal kernel especially for the offshore reefs of the NIWP.

The long-distance spawners, *T*. *gratilla* and *Epinephelus* sp., had similar mean distance of connections despite the latter having an age of settlement competency longer by seven days. The application of swimming behavior for the fish larvae demonstrated that even a conservative simulation of the reef detection radius could alter the connectivity results. Other studies [29,64] have acknowledged the role of larval behavior in modulating settlement and limiting the dispersal distance [69].

The range in the values of the settlement probabilities and the different connectivity matrices imply that the magnitude of connections in the NIWP is sensitive to early life history characteristics. However, the moderate to high correlations of metric values between organisms suggest that the relative spatial pattern of connectivity is more sensitive to the geographic configuration of reefs rather than the differences in dispersal potential of larvae.

### Sensitivity of connectivity to temporal variability

Through simulating connectivity during multiple seasons for each organism, this study demonstrated that the connectivity of the NIWP was sensitive to temporal variabilities. Correlations between seasonal matrices showed that the relative connectivity metrics were mostly similar between seasons suggesting once again the strong influence of the geographic location of reefs relative to seasonal circulation variabilities. The mean direction of connections of some areas were more sensitive to seasonal differences than others. If we were to focus on areas with high temporal variability, the actual spawning periods of organisms would be needed to increase the reliability of connectivity probabilities, especially if these probabilities were to be translated to biomass. Unfortunately, the actual spawning period of many coral reef organisms in the NIWP is often not known. For taxa that have been well studied like *Epinephelus* sp., the ranges of spawning periods vary highly within the region [55]. Furthermore, climate change may affect connectivity patterns [78] by offsetting the spawning timing of organisms that rely on seasonal changes in temperature [79–81] or circulation patterns [82,83]. This highlights the need for more studies on spawning periodicity and the effects of climate change on larval ecology in the NIWP.

Seasonal differences were more salient than interannual differences though this may partly be due to the use of only three representative years rather than a full ENSO cycle. Connection directions were more consistent across seasons for reefs at the eastern Luzon and eastern Mindanao because of the strong year-round boundary currents. Monsoonal reversals have been empirically demonstrated to induce variations in the direction of connectivity especially for longer PLD organisms [84]. The modelled seasonal variability implies that timing of spawning would be a critical factor in determining the connectivity of larval organisms [27], especially since even among fish species from the Philippines, spawning patterns are variable relative to the monsoons [55,84]. Year-round spawning would lead to interconnectedness, but this may be an energetically costly reproductive strategy. Spawning during a particular monsoon season would limit the range of connections and possibly isolate other areas. Spawning during the summer period, as exemplified by many coral species in the Philippines [53,85,86], may bear the advantage of having a wider range of possible connection directions since current magnitudes are weakest and least directed to a particular direction. The summer period seemed to be the average of the seasonal circulation patterns since summer matrices were more correlated with the annually-averaged matrices.

The interannual shifting of the NEC bifurcation latitude [37] may explain high interannual variability in the direction of connections at the Pacific-facing eastern Samar reefs where larvae may either be eventually transported by the Kuroshio or the Mindanao current. The NEC also influences Lamon Bay and Dinagat Sound [37] where sinks may either be more local due to retention or downstream of the western boundary currents. Additionally, interannual variability at the northwestern Lamon Bay may be related to the size and location of the cyclonic eddy modulated by the NEC [37]. Around the South China Sea, interannual differences in the formation of eddies especially during inter-monsoon periods may be the cause in shifts in the direction of connections for offshore and exposed reef sites. Interannual differences were also high around the Celebes Sea reflecting interannual variations in the Indonesian throughflow [46,87]. Determining the extent of interannual variabilities would be helpful for reefs that experience good or bad recruitment years [22,88–90].

### Management implications from cluster analysis

The clustering of reef cells according to covarying connectivity patterns is a novel method that can be used to delineate metacommunities for conservation and management. The BCD clustering algorithm, while only considering source and sink connections of each reef and not geographic distance, produced clusters defined by geographic positions and dominant circulation features (e.g. straits, bays, jets) [19]. These results point to the capacity of the BCD method to condense the variations inherent in usually large connectivity matrices. It can be useful in estimating which areas would similarly experience variabilities in larval supply due to the degradation, or conversely, recovery and enhancement of source reefs, as well as potential changes in connectivity directionality due to the mediation of climate modes to circulation patterns.

The level of clustering used would need to be informed by the spatial scale of interest. For example, in the Philippines, management of coral reefs is usually implemented at the level of local municipalities through community-based MPAs [91]. While the average cluster size of 9.7 ± 4 at MCS = 5 was well within the range of the mean number of reef cells of coastal municipalities (7.91 ± 5.7 reef cells per municipality) [92], partitioning of clusters did not necessarily correspond with political boundaries. The reef clusters not only synthesizes the connectivity information to a manageable resolution for pattern analysis and as inputs to decision-making tools [93], but also highlights the shared role of nearby municipalities in regional connectivity. Creating social networks between local management units encompassed by the different clusters would support ecological connectivity and potentially enhance management effectiveness [17,91].

Clustering at a coarse scale pointed out some general connectivity patterns between countries of the NIWP. The global connectivity model by Wood et al. [47] found that interregional connections have up to 10 times lower probabilities, and this seems to apply as well to transport between Philippine reefs and those of the rest of the NIWP. The circulation of the South China Sea appeared to be a barrier that limits potential exchange of larvae between the Philippines and mainland Southeast Asia. Connections at the central South China Sea were mostly seasonal current reversals between Vietnam and the Paracel Islands with the Spratly Islands and northwestern Luzon. Reefs near the Karimata Strait rarely connected directly to the southern Palawan and Spratly Islands because of the general southward flow near the strait. Although the Philippines may be an upstream larval source to the surrounding countries [47], the low probabilities of these potential connections are likely not enough to affect the demographics of the sink reefs [25,90]. Regardless, clusters spanning multiple countries were found, including those around northern Borneo and the Sulu Archipelago and of the Celebes Sea. This emphasizes the value of international partnerships in coral reef management given how these areas share similar regional roles in supplying larvae to the region.

### Concordance of model results with empirical observations

The location of inferred barriers to gene flow summarized in Von der Heyden et al. [94] appears to be concordant with the implied limitations of the spatial extent of larval exchange. In the model, northeastern Luzon was the most isolated of Philippine reefs. This is likely because it is downstream of the persistent NEC and has a limited range of sources. This region was also found to house genetically distinct populations of *Tridacna crocea* [95], *Chanos chanos* [96], and *Siganus fuscescens* [97]. Observed differences in the populations in the northwestern South China Sea, Gulf of Thailand, Karimata Strait, and the Philippines [94] can be explained by the limited larval exchanges modelled between these reefs. Contrastingly, the modelled high interconnectivity between the Spratly Islands and western Palawan [98] reflects the strong gene flow detected across these reef sites. The perceived distinction of the Philippine internal seas [94] is concordant with the high likelihood of retention simulated around the Visayan and Sibuyan Seas, and the limited range of interconnections of the internal reefs with the Sulu Sea. The north-south genetic distinction of the Celebes Sea [94] differentiating the Philippine and Indonesian sides may likely be a result of the year-round southward throughflow and the eddy dipole produced by the Mindanao current [99] resulting to limited connections from southern to northern reefs. The genetic partitioning that grouped the western Philippines, Sulu Sea, northern Celebes Sea, Bohol Sea, and eastern Mindanao as one population cluster [94] was likely a result of the high interconnectivity of reefs of these regions.

The spatial match between the modelled regional connectivity patterns with inferred genetic barriers provides a coarse validation of large-scale exchange of larvae in the NIWP. Validation with population structure is still insufficient for finer-scale spatial patterns especially since genetic and ecological interconnectedness can be mutually exclusive, i.e., populations that are evolutionarily linked may be demographically separated [25]. The clustering of reefs around the Bohol Sea did not strictly agree with the connectivity clusters based on species assemblage demonstrated by Abesamis and colleagues [100], although their study better resolved the Bohol Sea coastline. At the mesoscale, the hydrodynamic data used in this study was consistent with oceanographic observations [38,50,101] assuring that the transport mechanisms yielding the patterns of reef directionalities were reliable in terms of time-averaged circulation patterns.

Recent studies on parentage analyses of various reef organisms provide optimistic empirical demonstrations of demographic connectivity at both local and regional scales. Parent-juvenile pairings have shown both greater local retention at the natal reef or reef region and more importantly, long-distance connections representing the tail of dispersal up to distances of 48 km to 400 km for different species [21,82,100–106]. The estimates of the dispersal distances of this study are within the estimated empirical distances from fitted dispersal kernels with 95% (50%) of dispersal at 83 km (33 km) for *C*. *vagabundus* [84], and 480 km (110 km) to 811 km (185 km) for *P*. *maculatus* and *P*. *leopardus* [106], respectively. These empirical estimates of connectivity suggest that the long-distance regional connectivity modelled in this study likely occurs [106] and is regular rather than rare and stochastic [105].

### Recommendations for model development

The model was only able to account for surface flow and near-surface reefs. Subsurface pathways that likely retained more larvae near the natal reef would best be resolved with three-dimensions. Such a modelling approach could potentially capture depth variability of spawning and settlement, especially for reef slopes and mesophotic reefs [33,107], together with the capability of many larvae to regulate their vertical position in the water column [41,108]. Larvae of brooding organisms, which are likely negatively buoyant and could readily settle, were likewise not represented in the model. Although the parameters used for settlement age and PLD are representative of the usual range of larval reef organisms, longer PLDs and onset of settlement have already been observed [19,69,103]. Most importantly, a more comprehensive set of ecological functions like realistic mortality scenarios, the effect of reef health and cover in larval supply and settlement, and post-settlement scenarios were excluded from the model to focus on determining potential connectivity patterns. It would be interesting to incorporate these in future simulations of the model.

Improving the model results would primarily entail (1) increasing the resolution which would resolve the narrow straits and complex coastlines and (2) coupling larval dispersal with a population growth model using realistic estimates of larval supply and settlement habitat quality. Applying the connectivity results to matrix projections [31] and seascape genetics [109–111] would better link demographic connectivity with population genetics studies. It would be interesting to numerically test how much larval dispersal explains population genetic diversity using the biophysical connectivity estimates from this study.

## Conclusions

Our simulation of the larval connectivity of three coral reef organisms demonstrated that connectivity is inherently variable in a dynamic region like the NIWP. Both early-life history characteristics and temporal differences in circulation resulted in variations in the magnitudes of the settlement probabilities and connectivity metrics. However, based on the generally moderate to high correlations of these metrics between organisms and between time periods, connectivity in the NIWP seems more sensitive to the geographic configuration of reefs compared to differences in dispersal potential of larvae or circulation. Concordance of the modeled ranges of dispersal with the range of the empirical estimates from parentage analyses, and the identification of clusters of co-varying connectivity patterns with population genetic studies, provide support for the validity of the model results.

Apart from illustrating the mesoscale patterns in larval connectivity for the NIWP, we offer three tools which could be used to bridge connectivity from the global to local levels for the NIWP: (1) fitted dispersal kernel functions providing estimates of the relative probabilities of connection given only the distances between reefs; (2) maps on the relative sensitivity of different reefs to temporal connectivity variability; and, (3) the clustering of reefs using the BCD algorithm, which produced clusters defined by the geographical position of reefs relative to dominant circulation patterns and features. These clusters of co-varying connectivity patterns can serve as inputs into efforts for the conservation and management of NIWP coral reefs from regional to local levels.

## Supporting information

S1 Appendix Model overview, design concepts, and details. (DOCX)

S2 Appendix Model calibration and sensitivity analysis. (DOCX)

S3 Appendix Tables of Post-hoc Dunn’s test. (DOCX)

S4 Appendix Figures on temporal differences in metrics and direction distributions. (DOCX)

S5 Appendix Figures on the clusters formed at other MCS values. (DOCX)
