# Supplementary material for: Larval connectivity patterns of the North Indo-West Pacific coral reefs
Source: PLoS One. 2019 Jul 23;14(7):e0219913. doi: 10.1371/journal.pone.0219913 (PMC6650046; doi:10.1371/journal.pone.0219913)
Supplement: S1 Appendix — (DOCX) [file pone.0219913.s001.docx]

**1. Purpose**

The model aimed to simulate the connectivity of larvae between coral reefs. It was designed to be similar to other larval dispersal models like the Connectivity Modelling System [1] and was especially coded under the Java-Mason platform [2] to include a horizontal swimming behavior module, settlement probability module, and a model output scheme which utilized less storage space

**2. Entities, State Variables, and Scales**

**2.1. Environment**

The model environment was a set of unstaggered Arakawa-A grids with a size of 363 x 309. Four grids contain the u-component and v-component of current vectors for two successive days. A land masking grid was marked 0 for land and 1 of water. A grid identifying coral reef locations was numbered sequentially from 1 to 3776 for all coral reefs cells and zero otherwise. A continuous grid of the same size contains all the model agents.

The spatial scale of the model is 0°N to 24°N and 99°E to 128°E with a resolution of 0.08°. The current grids were set to update at daily intervals. The temporal span possible is from November 2008 to October 2016 corresponding to the available global HYCOM dataset.

**2.2. Agents**

The model agents represented different types of larvae. All agents were marked with a source reef variable consistent with the reef cell code the agent was initialized on. Agents have a spatial position in both geographic coordinates (degrees) and model units which updates every time step. Agents have an age variable measured in days. The parameters which distinguishes the larval agents were age of settlement competency, pelagic larval duration (PLD), mortality rate, and swimming behavior. If swimming was applied to the agent, the agent had a flexion age and swimming speed (cms^-1^) which was a function of its age in days.

**3. Process Overview and Scheduling**

The model begins by creating and initializing the different environmental grids. The data files on land masking and coral reef cell locations are loaded into their respective grids. The model then reads the parameters for the agents and seeds the agent on the continuous environment grid according to the agent density per coral reef cell.

The model runs at a discreet time step of 2,700 seconds. During the first time step of each model day, the current vector data are loaded to their respective grids. During each time step, the spatial location of model agents is updated in random order following the movement submodel. If the agent is still inside the model domain, the probability of mortality is computed. If the agent would die, it is stopped and removed from the environment. If the agent is still alive, settlement will be evaluated based on the competency and settlement submodel. If the agent would settle, the source cell and sink cell information will be used to update the connectivity matrix. After the end of each model day, the age of the agent is increased.

Once the model reaches its maximum running day, the connectivity matrix will be outputted into a comma-separated file. The model would terminate afterwards.

**4. Design Concepts**

**4.1. Basic Principles**

The underlying principle of the model is the dispersal of planktonic larvae of coral reef organism which is predominantly influenced by ocean circulation [3]. The settlement of larvae follows a competency approach in which the larval agent can settle starting on the settlement onset day until PLD [4]. Swimming behavior by the agents is based on the ability of some larvae to sense and direct their swimming towards coral reefs [5].

**4.2. Emergence**

The spatiotemporal distribution of agents emerges from the effect of the current forcing and swimming behavior if applicable. It is modulated by the mortality of the agents. The ultimate emergent pattern is the larval connectivity recorded in the connectivity matrix which results from the stochastic interactions between the agent’s location and its competency for settlement.

**4.3. Adaptation**

Adaptive capabilities are only applicable for simulated fish larvae which are able to swim towards the nearest coral reef within a one-cell radius.

**4.4. Objective**

The objective of the agent is to position itself to a coral reef cell either passively or actively and settle onto a reef cell.

**4.5. Learning**

Learning by agents is not applied in the model.

**4.6. Prediction**

Prediction by agents is not applied in the model.

**4.7. Sensing**

Agents can identify if the cell they are located on is a coral reef cell or not. Simulated fish larvae can sense coral reef cells within a one-cell radius.

**4.8. Interaction**

Only the model environment varies the state variables of the agents. The agents behave independently from other agents. Density-dependent interactions like competition for space are not included.

**4.9. Stochasticity**

A random number generator is used during the seeding of agents in the initialization stage of the model where agents are randomly seeded within a grid cell. During every time step, agents are scheduled at a random order before undergoing the different submodels.

In the agent movement submodel, a random number between -1 to 1 is used in the computation for dispersion due to turbulence in the variable. Stochasticity is also applied to determine if the agent would die or settle during each time step.

In applying the swimming behavior, if the larva is located equally distant to two surrounding reef cells, it randomly selects one to swim towards.

**4.10. Collectives**

Collectives of agents are not considered in the model.

**4.11. Observation**

An omniscient observation produces a tally of all agents which have settled based on their sink and source reef cell.

**5. Initialization**

The model initializes during midnight of a specific date. The end date of the model is also concurrently set. The different environment grids are then created and initial data are loaded. Agents are randomly seeded in each coral reef cell based on a predetermined larva per cell density. The agent’s initial location and source reef cell code is assigned for each agent and the age is set to zero. If the agent is a fish larvae, its swimming behavior is initially turned off.

**6. Input Data**

The hydrodynamic data used in the model is the global Hybrid Coordinate Ocean Model (HYCOM) [6,7] GLBa0.08 surface circulation. The HYCOM land mask is also used in the model. Coral reef cells were determined based on the UNEP World Conservation Monitoring Center (UNEP-WCMC) coral reef database [8] after a GIS intersection with model land masking. Parameters used for each agent are enumerated in the table below.

Table 1. Parameters differentiating each model organism.

| **Organism** | **Age of settlement competency (days)** | **Maximum PLD (days)** | **Mortality rate (day^-1^) *** | **Swimming behavior** |
| --- | --- | --- | --- | --- |
| *Acropora millepora* | 3 [9] | 60 [9] | 0.023 | No |
| *Tripneustes gratilla* | 29 [10] | 57 [11] | 0.024 | No |
| *Epinephelus* sp. | 36 [12] | 47 [12] | 0.029 | Yes** |

* derived from a half-life equation [4,13]

** sustained swimming speed was derived as 50% [14] of the critical swimming speed as a function of age.

**7. Submodels**

**7.1. Movement Submodel**

Movement of the agents is a result of advection, diffusion, and swimming. Advection is the transport of agents due to currents and is computed by deriving the instantaneous current vectors at the exact location of each agent. Since HYCOM circulation data are available at daily intervals and spaced 0.08° apart, the currents value will be computed by bilinear interpolation across space considering the four nearest data points and linear interpolation across time with each data log assumed to occur during the midnight of each day. The advection of larvae will use a Runge-Kutta 4th order differential equation scheme which models particle transport more realistically especially near land boundaries [15].

Diffusion is a random-walk equation given by,

$Diffusion= \gamma\sqrt{\Delta t \cdot K}$, (1)

where γ is a random number from (-1 to 1), is the model time interval, and D is the diffusivity coefficient. The value of K is 7.25 m^2^s^-1^ estimated from an assumed diffusion length scale of 1/12° [16] which is the resolution of the hydrodynamic model. This is similar to the value of K in Wood et al. [4] with a similar model resolution.

For the fish larvae, horizontal swimming is applied once reaching the flexion age set at 20 days [17]. Each post-flexion *Epinephelus* sp. larvae searched the adjacent grid cells relative to its current position. If reef cells were detected, distances towards the adjacent reef cells was computed and swimming was directed towards the nearest reef cell. The sustained swimming speed was computed as 50% of the critical swimming speed [14] which was derived from an age–swimming speed function [18]. SSIf no reef cells are detected, the swimming module is disabled for the current time step. Once inside a reef cell, swimming is also disabled. This method in modelling larval swimming behavior is based on Wolanski and Kingsford [5]. When a larva encounters a land boundary, it will be reflected to the ocean cell at the angle of incidence. If the larva is transported beyond the model domain, the larva is removed from the simulation.

**7.2. Competency and Settlement Submodel**

Larval mortality will be computed at the end of each model day by randomly drawing a value from 0 to 1 for each larva. If this is below or equal to the mortality rate of the organism (Table 1), the larva is considered dead and removed from the simulation. Upon reaching the maximum pelagic larval duration (PLD) and not on a suitable habitat, larvae are then considered dead [3] and removed from the simulation.

The ability to settle will be based on the age of competency. Once larvae are competent and located on a reef cell, settlement can occur during any time step. The probability of settlement will be set to a baseline of 0.5 similar to Dorman et al. [19]. This would mean that some competent larvae can pass by a reef cell without settling on it. This attempted to simulate the continuous effect of advective and diffusive forces while a larva is moving towards the benthic reef structure. Once larvae settle, the current reef cell would be considered as the sink cell. The source and sink reef cell codes are recorded into the connectivity matrix and the larvae would then be removed from the model.

# References

1. Paris CB, Helgers J, Van Sebille E, Srinivasan A. Connectivity Modeling System: A probabilistic modeling tool for the multi-scale tracking of biotic and abiotic variability in the ocean. Environ Model Softw. 2013;42: 47–54.

2. Luke S, Cioffi-Revilla C, Panait L, Sullivan K, Balan G. Mason: A multiagent simulation environment. Simulation. 2005;81: 517–527.

3. Cowen RK, Sponaugle S. Larval dispersal and marine population connectivity. Annu Rev Mar Sci. 2009;1: 443–66.

4. Wood S, Paris CB, Ridgwell A, Hendy EJ. Modelling dispersal and connectivity of broadcast spawning corals at the global scale. Glob Ecol Biogeogr. 2014;23: 1–11.

5. Wolanski E, Kingsford MJ. Oceanographic and behavioural assumptions in models of the fate of coral and coral reef fish larvae. J R Soc Interface. 2014;11: 20140209.

6. Bleck R. An oceanic general circulation model framed in hybrid isopycnic-Cartesian coordinates. Ocean Model. 2002;4: 55–88.

7. Chassignet EP, Hurlburt HE, Smedstad OM, Halliwell GR, Hogan PJ, Wallcraft AJ, et al. The HYCOM (hybrid coordinate ocean model) data assimilative system. J Mar Syst. 2007;65: 60–83.

8. UNEP-WCMC T. Global distribution of warm-water coral reefs, compiled from multiple sources including the Millennium Coral Reef Mapping Project. UNEP World Conservation Monitoring Centre Cambridge, UK; 2010.

9. Connolly SR, Baird AH. Estimating dispersal potential for marine larvae: dynamic models applied to scleractinian corals. Ecology. 2010;91: 3572–3583.

10. Juinio-Meñez MA, Hapitan RM. Mass culture of the sea urchin *Tripneustes gratilla.* Transactions of the National Academy of Science and Technology Proceedings of the 20th Annual Scientific Meeting and the 4th National Social Science Congress of the Department of Science and Technology, July. 1998. pp. 8–9.

11. Juinio-Meñez MA, Bangi HGP. Extrinsic and intrinsic factors affecting the metamorphic rate of *Tripneustes gratilla* (Echinodermata: Echinoidea). Mar Ecol Prog Ser. 2010;402: 137–145.

12. Alava MNR, Dolar MLL, Luchavez JA. Natural spawning of four Epinephelus species in the laboratory. Proceedings of the Seminar-Workshop on Breeding and Seed Production of Cultured Finfishes in the Philippines, Tigbauan, Iloilo, Philippines, 4-5 May 1993. SEAFDEC Aquaculture Department; 1996. pp. 65–77.

13. Sponaugle S, Paris C, Walter KD, Kourafalou V, Alessandro ED. Observed and modeled larval settlement of a reef fish to the Florida Keys. Mar Ecol Prog Ser. 2012;453: 201–212.

14. Fisher R, Leis JM. Swimming Speeds in Larval Fishes: From Escaping Predators to the Potential for Long Distance Migration. In: Domenici, P, Kapoor BG, editors. Fish locomotion : an eco-ethological perspective. Enfield, NH: Perspective Science Publishers; 2010. pp. 333–373.

15. North EW, Gallego A, Petitgas P. Manual of recommended practices for modelling physical–biological interactions during fish early life. ICES Coop Res Rep. 2009;

16. Okubo A. Oceanic diffusion diagrams. Deep sea research and oceanographic abstracts. Elsevier; 1971. pp. 789–802.

17. Sabate F de la S, Sakakura Y, Shiozaki M, Hagiwara A. Onset and development of aggressive behavior in the early life stages of the seven-band grouper *Epinephelus septemfasciatus*. Aquaculture. 2009;290: 97–103.

18. Leis JM, Hay AC, Lockett MM, Chen J-P, Fang L-S. Ontogeny of swimming speed in larvae of pelagic-spawning, tropical, marine fishes. Mar Ecol Prog Ser. 2007;349: 255–267.

19. Dorman JG, Castruccio FS, Curchitser EN, Kleypas JA, Powell TM. Modeled connectivity of *Acropora millepora* populations from reefs of the Spratly Islands and the greater South China Sea. Coral Reefs. 2016;35: 169–179.
