# Supplementary material for: Larval connectivity patterns of the North Indo-West Pacific coral reefs
Source: PLoS One. 2019 Jul 23;14(7):e0219913. doi: 10.1371/journal.pone.0219913 (PMC6650046; doi:10.1371/journal.pone.0219913)
Supplement: S2 Appendix — (DOCX) [file pone.0219913.s002.docx]

# **Calibration**

Model calibration and sensitivity analysis utilized a subset of 100 randomly chosen reef cells (Fig A). Calibration was first done on the number of larvae to be spawned for each model organism and the temporal intervals of spawning or initiation of model simulations as these parameters are of primary concern for dispersal modelling [1,2]. The coarsest possible parameter values that would limit the necessary computing power and data output storage sizes were determined by using the field of unexplained variance (FUV) method [2]. In this method, different parameter values were compared to the finest feasible values that serves as the baseline values. FUV threshold was set to 0.05 meaning that the output of the tested value was similar (i.e., correlated) by 95% to the baseline. The baseline values were 10,000 larvae spawned per cell and a daily spawning interval. Simulations were made during the 91^st^ day of each model year. The outputs compared were all source to sink connections present in either the baseline connectivity matrix or the matrix resulting from the calibrated value.

The selected parameter values of 100, 250, and 450 larvae per cell for *Acropora millepora*, *Tripneustes gratiilla*, and *Epinephelus* sp., respectively, were all well below the FUV threshold (Fig B) and further increasing the value relative to these still led to the FUV remaining under 0.05. These values were close to that of Holstein et al. [3] which had a similar model resolution and set of larval parameters.

Connectivity matrices from daily spawning simulations were resampled at every integer interval until 30 days and averaged across time. A spawning interval of every five days produced mean connectivity matrices which were similar to the matrix of the baseline daily spawning averaged over 90 days for all model organisms (Fig C) thus this was the parameter value used for the main model runs. This model seeding rate is more frequent than the monthly seeding of other connectivity models [4,5].


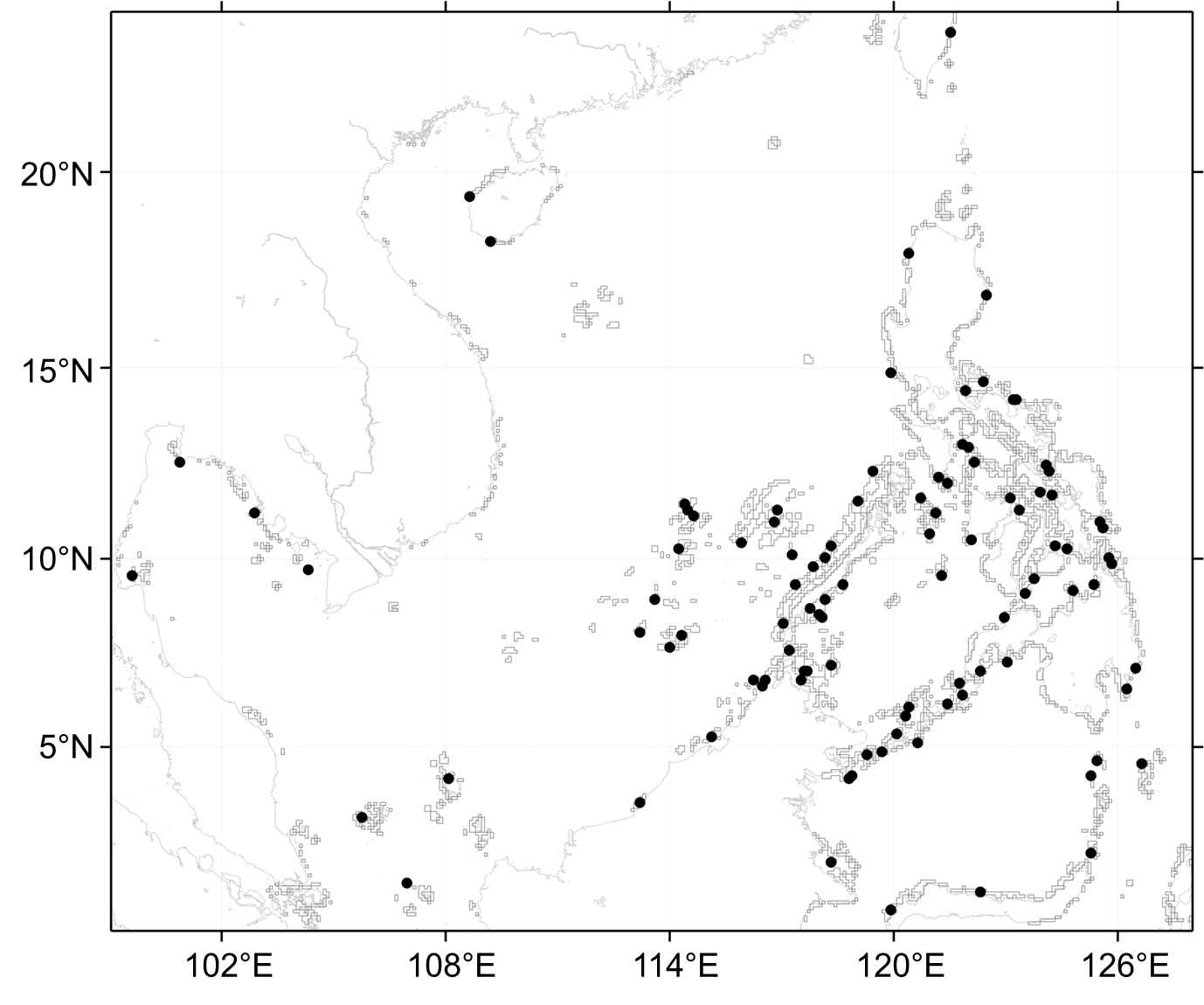


Figure A. Map of 100 randomly chosen larval release cells (dots) for calibration and sensitivity analysis. Polygons mark coral reef areas.


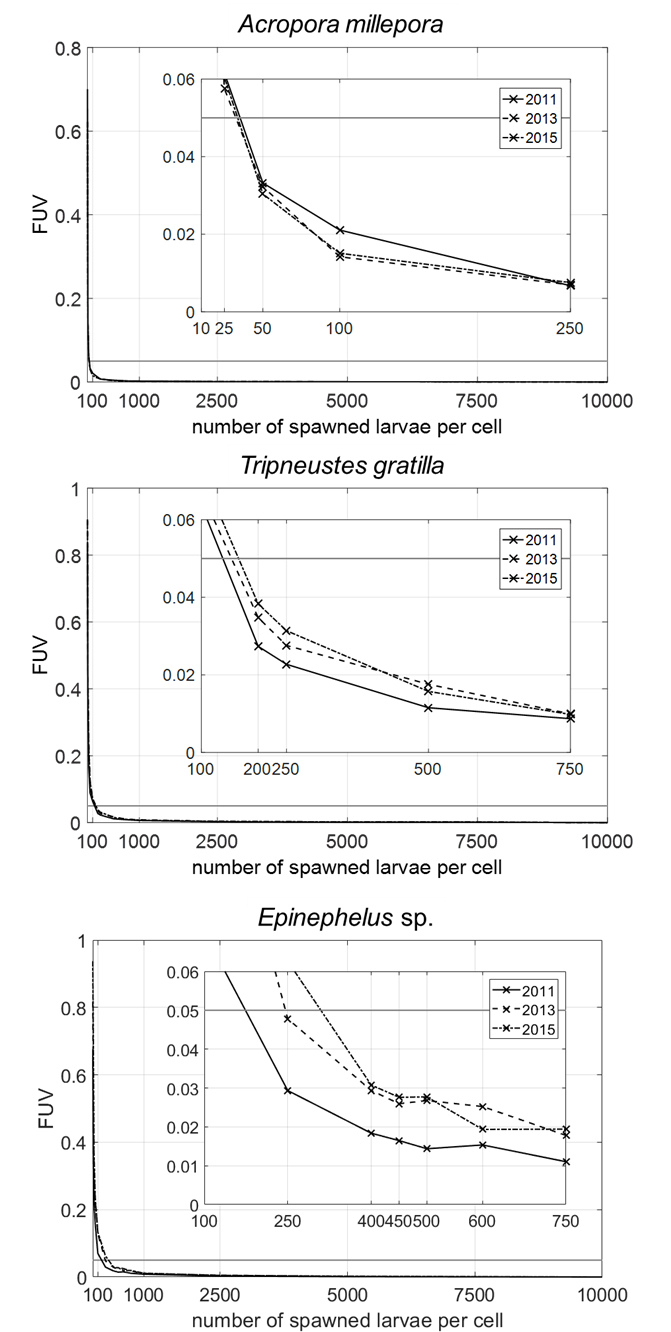


Figure B. Calibration of the number of spawned larvae per cell. Inset boxes are zoomed in at ranges close to the FUV threshold (solid horizontal line). The selected values of 100, 250, and 450 for *Acropora millepora*, *Tripneustes gratiilla*, and *Epinephelus* sp. produced similar connectivity matrices to simulations with 10,000 larvae per cell.


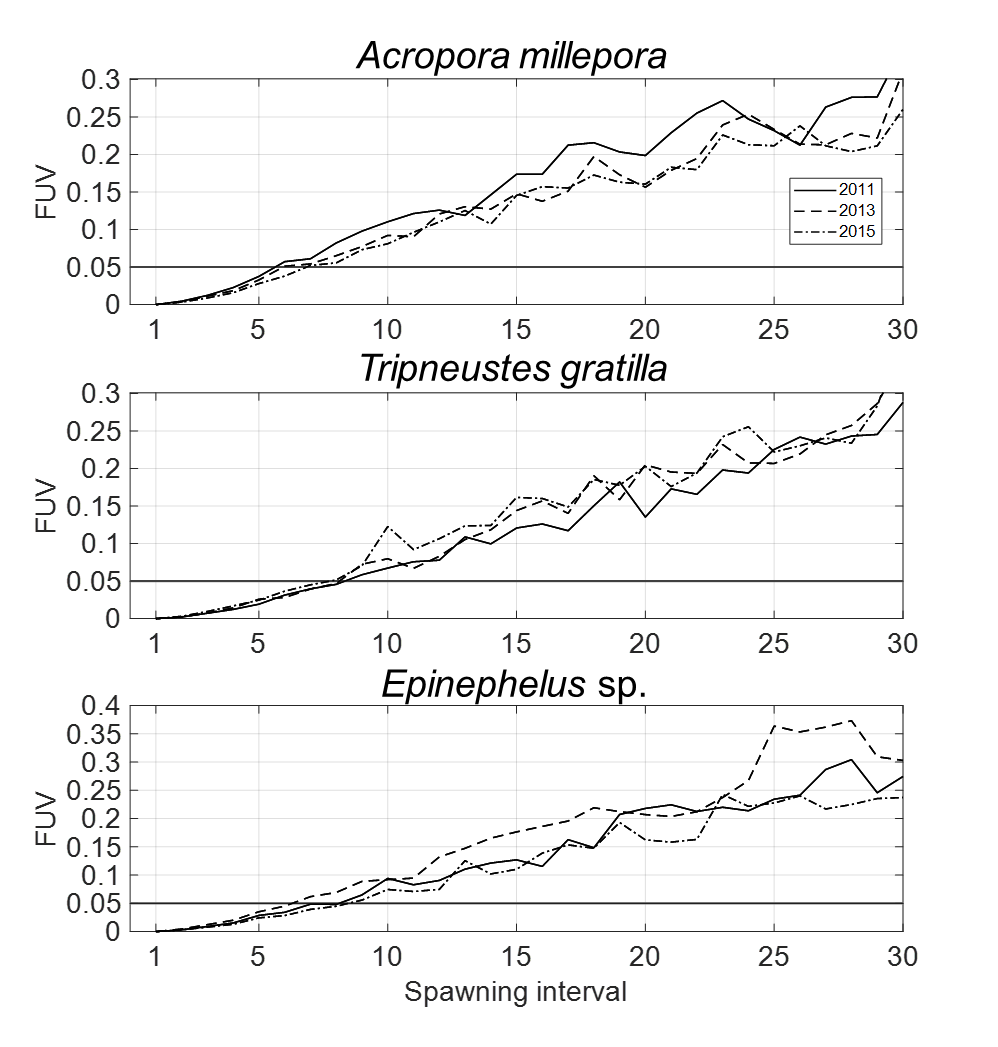


Figure C. Calibration of the spawning interval. Intervals below the FUV threshold (solid horizontal line) were similar relative to the connectivity matrix based on daily spawning for 90 days. The spawning interval of 5 days was selected for all organisms.

# **Sensitivity Analysis**

Sensitivity analyses of the biological parameters also utilized the FUV method in which the baseline values were determined from literature with test values diverging from the baseline at regular intervals (Table A). The objective was to identify with-in organism model sensitivity. Sensitivity scenarios were initiated during the 91^st^ day of each model year by spawning the determined number of larvae per cell from the calibration at the 100 random reef cells.

All model organisms were not sensitive to varying the PLD value (Fig D) which was the maximum tracking days of the model organisms. After even less than 10 days from the representative settlement ages, the connections formed did not significantly change the connectivity matrix suggesting that most of the modelled settlement occurred a few days after the onset of settlement competency. A benefit of extending the model simulation up until the actual determined PLD was allowing rare connections which capture the potential spatial extent of connectivity.

The mortality rate was a half-life function of the PLD assuming an inverse relationship. *Acropora millepora* was not sensitive to varying the mortality rate (Fig E) because the early onset of settlement competency allowed forming connections well before a substantial proportion of the larvae have been removed from the model. Both *T. gratilla* and *Epinephelus* sp. were not sensitive to small variations of the mortality rate up until a 25% increase from the baseline while a 50% increase resulted to FUV values beyond the threshold. This increase resulted to greatly reducing the modelled larval pool even before reaching settlement age. It should be noted that the mortality rate applied in this model does not strictly correspond to the actual natural mortality rate but was a modelling method done to prevent overloading the model with larvae only to greatly reduce them during the first few time steps.

Settlement age was determined to be the most critical biological parameter (Fig F) that varying this value by just one day changed the connectivity matrix by more than 5% to around 40%. Advection for two days with currents as weak as 0.1 ms^-1^ could transport larvae across three model cells thus changing the extent of larval dispersal. Current magnitudes are typically far greater for the open ocean and straits. Thus, comparing the modelled organisms to specific taxa should initially caution on the settlement onset age. In terms of this study providing a range of biological parameters, it was reasonable to expect that between-model organism differences would be largely explained by settlement age.

The genetic structure (F_ST_) of populations is the result of gene flow largely attributed to larval dispersal and theoretically, higher dispersal potential leads to weaker structures [6]. The lack of or weak correlation between PLD and F_ST_ noted by multiple comparative studies [7–10] have placed dispersal potential under scrutiny of being an effective predictor of population structures. This sensitivity analysis suggests that genetic connectivity may find a better correlation with the onset of settlement competency [11]. Settlement age may thus be a more appropriate parameter to prioritize in models to distinguish larvae and predict the possible spatial range of connections. PLD may be more relevant to compare brooders [10,12,13] or early-onset spawners with long-distance spawners [14] hinted by different levels of population structure of two Pocilloporidae species attributed to their dispersal abilities [15].

An arbitrary settlement probability of 0.5 was set for the main model simulations following Dorman et al. [16] to simulate the possibility of larvae being continuously advected by currents as it settles towards the benthos. Model results were not sensitive to this parameter (Fig G) due to repeated attempts of settlement still occurring on the same reef cell given the fine temporal resolution of the model. This would thus be almost equivalent to having a settlement probability of 1.0 which is typically used in most connectivity models.

The simulated ability of *Epinephelus* sp. larvae to swim towards the reef one model cell away varied the output by more than 25% upon comparing the flexion age of 20 days [17] to that of the PLD of 47 days which practically simulates no swimming behavior (Fig H). Even setting the flexion age 2 days before the PLD resulted to FUV breaching the threshold. Flexion age relative to 20 days was not as sensitive to variations by around ±5 days (Fig H) as swimming speeds were still considerably weaker at these ages compared to the ambient current magnitudes. Thus, the period when swimming greatly changed the trajectory of the modelled fish larvae was more than 10 days after flexion age around the time when the fish larvae may begin to settle.

The sustained swimming speed of *Epinephelus* sp. larvae was estimated [18] as a fraction of the critical swimming speed (U-crit). The recommended value [18] that we applied in the main model is 50%. The model was sensitive to the speed of active swimming that variations of at least 10% significantly changed the model output (Fig I). Simulating no swimming, at 0% of U-crit, had a high FUV compared to the standard 50% U-crit. This suggests that the recommended estimate of sustained swimming speed would still be effective in simulating the active swimming capabilities of *Epinephelus* sp. larvae.

Table A. Sensitivity analysis parameter values for each model organism.

| **Parameter** | ***Acropora millepora*** | ***Tripneustes gratilla*** | ***Epinephelus* sp.** |
| --- | --- | --- | --- |
| PLD (days) | 4, 10, 20, 30, 40, 50, 55, 56, 57, 58, 59, **60***, 61, 62, 63, 64, 65, 70 | 30, 40, 50, 52, 53, 54, 55, 56, **57***, 58, 59, 60, 61, 62, 70 | 37, 40, 42, 43, 44, 45, 46, **47***, 48, 49, 50, 51, 52, 60, 70 |
| Mortality Rate | 0, 0.0115, 0.0173, 0.0208, 0.0220, 0.0229, **0.0231***, 0.0233, 0.0243, 0.0254, 0.0289, 0.0347, 0.0462, 0.0693 | 0, 0.0123, 0.0182, 0.0219, 0.0231, 0.0241, **0.0243***, 0.0246, 0.0255, 0.0268, 0.0304, 0.0365, 0.0486, 0.0730 | 0, 0.0147, 0.0221, 0.0265, 0.0280, 0.0292, **0.0295***, 0.0298, 0.0310, 0.0324, 0.0369, 0.0442, 0.0590, 0.0885 |
| Age of Settlement Competency (days) | 1, 2, **3*,** 4, 5, 6, 7, 8, 9, 10, 11, 12 ,13, 14, 15, 16, 17, 18, 19, 20, 21, 22, 23, 24, 25 | 14, 15, 16, 17, 18, 19, 20, 21, 22, 23, 24, 25, 26, 27, 28, **29***, 30, 31, 32, 33, 34, 35, 36, 37, 38, 39, 40, 41, 42, 43 | 18, 19, 20, 21, 22, 23, 24, 25, 26, 27, 28, 29, 30, 31, 32, 33, 34, 35, **36*,** 37, 38, 39, 40, 41, 42, 43, 44, 45, 46, 47 |
| Settlement Probability | 0.05, 0.25, **0.50***, 0.75, 1.0 | | |
| Flexion Age (days) | n.a. | n.a. | 16, 17, 18, 19, **20*,** 21, 22, 23, 24, 25, 30, 35, 40, 45, 46, **47*** |
| Ratio of Swimming Speed Relative to Critical Swimming Speed | n.a. | n.a. | 0, 0.1, 0.2, 0.3, 0.4, **0.5*,** 0.6, 0.7, 0.8, 0.9, 1.0 |

***** baseline value in FUV analysis


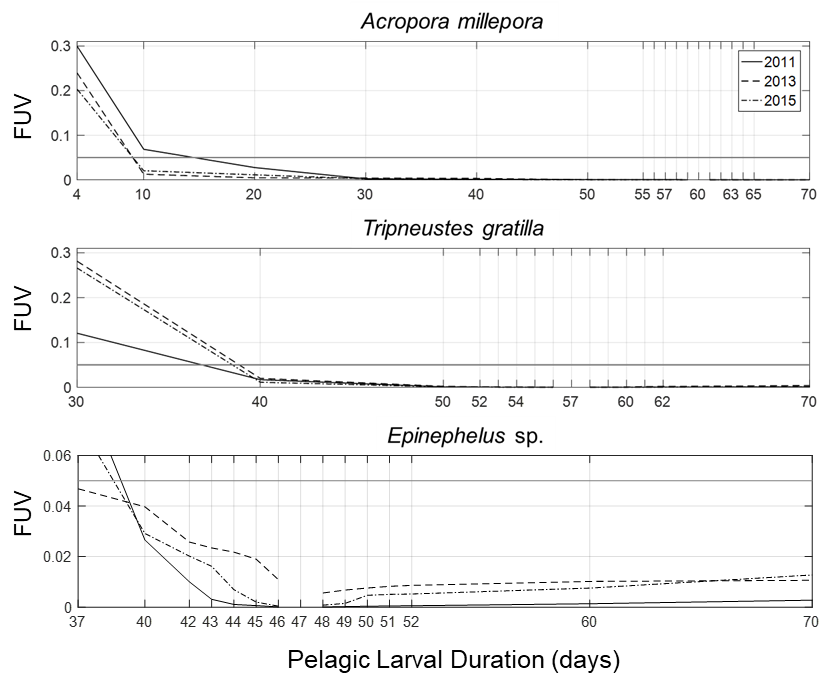


Figure D. Sensitivity analysis of the pelagic larval duration parameter.


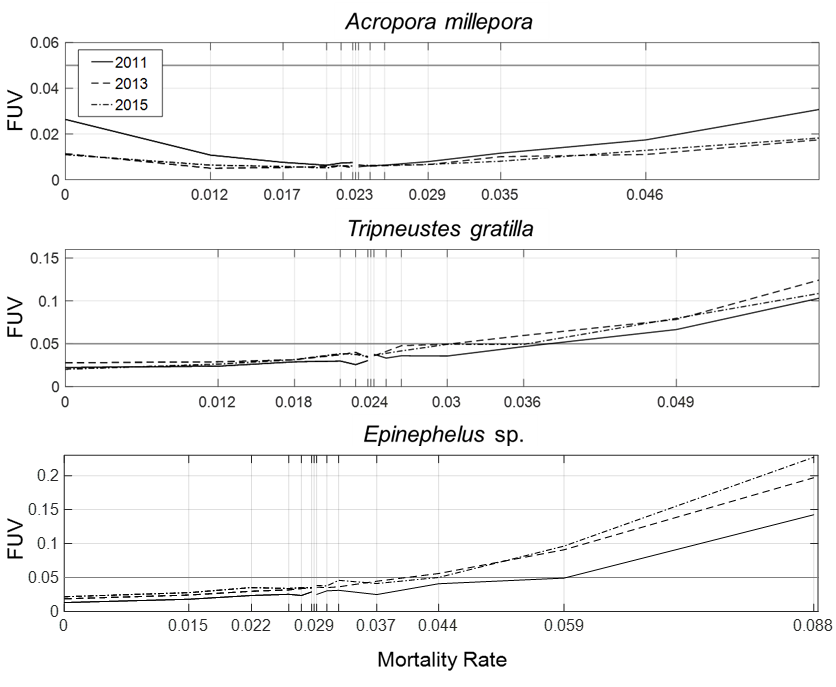


Figure E. Sensitivity analysis of the mortality rate parameter.


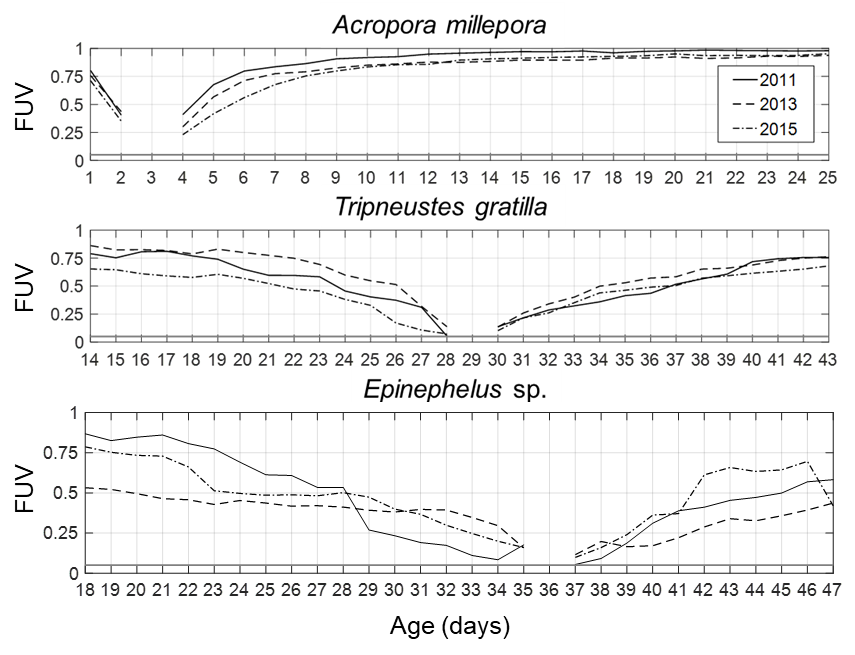


Figure F. Sensitivity analysis of the age of settlement competency parameter.


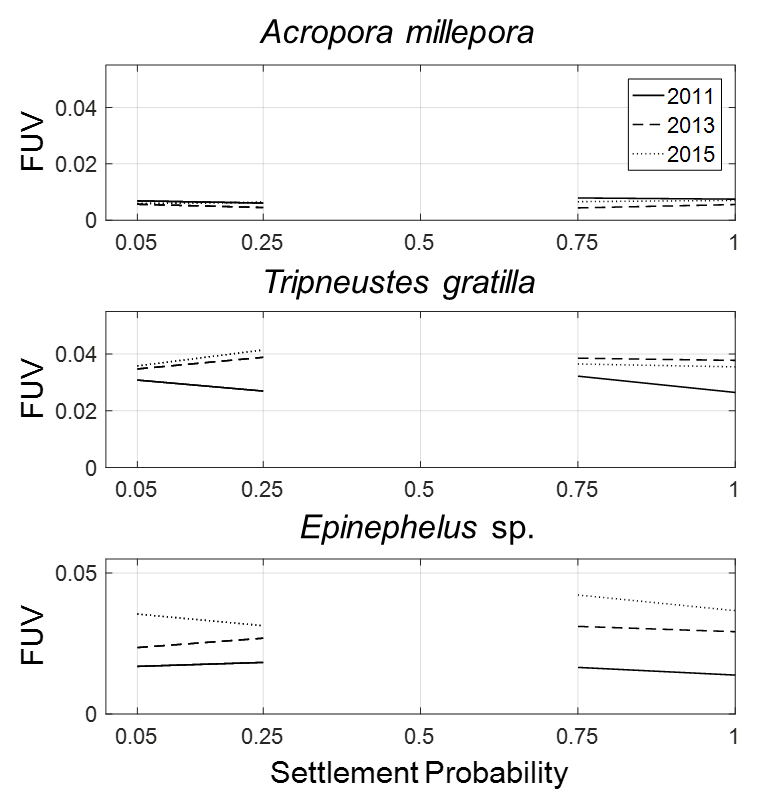


Figure G. Sensitivity analysis of the settlement probability parameter. All values were below the FUV threshold

Figure H. Sensitivity analysis of the flexion age parameter for *Epinephelus* sp. The baseline matrix for the figure above has a flexion age at 47 days which simulates no swimming. The figure below has a flexion age at 20 days based on literature.

Fig I. Sensitivity analysis of the estimated sustained swimming speed a fraction of the critical swimming speed (U-crit). The baseline matrix for the figure above is 50% of U-crit. The figure below is relative to U-crit.

**References**

1. Kough AS, Paris CB. The influence of spawning periodicity on population connectivity. Coral Reefs. 2015;34: 753–757.

2. Simons RD, Siegel DA, Brown KS. Model sensitivity and robustness in the estimation of larval transport: a study of particle tracking parameters. J Mar Syst. 2013;119: 19–29.

3. Holstein DM, Paris CB, Mumby PJ. Consistency and inconsistency in multispecies population network dynamics of coral reef ecosystems. Mar Ecol Prog Ser. 2014;499: 1–18.

4. Wood S, Paris CB, Ridgwell A, Hendy EJ. Modelling dispersal and connectivity of broadcast spawning corals at the global scale. Glob Ecol Biogeogr. 2014;23: 1–11.

5. Kool JT, Paris CB, Barber PH, Cowen RK. Connectivity and the development of population genetic structure in Indo-West Pacific coral reef communities. Glob Ecol Biogeogr. 2011;20: 695–706.

6. Faurby S, Barber PH. Theoretical limits to the correlation between pelagic larval duration and population genetic structure. Mol Ecol. 2012;21: 3419–3432.

7. Bowen BW, Bass AL, Muss A, Carlin J, Robertson DR. Phylogeography of two Atlantic squirrelfishes (Family Holocentridae): exploring links between pelagic larval duration and population connectivity. Mar Biol. 2006;149: 899–913.

8. Kelly RP, Palumbi SR. Genetic structure among 50 species of the northeastern Pacific rocky intertidal community. PLoS One. 2010;5: e8594.

9. Riginos C, Douglas KE, Jin Y, Shanahan DF, Treml EA. Effects of geography and life history traits on genetic differentiation in benthic marine fishes. Ecography. 2011;34: 566–575.

10. Weersing K, Toonen RJ. Population genetics, larval dispersal, and connectivity in marine systems. Mar Ecol Prog Ser. 2009;393: 1–12.

11. Connolly SR, Baird AH. Estimating dispersal potential for marine larvae: dynamic models applied to scleractinian corals. Ecology. 2010;91: 3572–3583.

12. Harii S, Kayanne H, Takigawa H, Hayashibara T, Yamamoto M. Larval survivorship, competency periods and settlement of two brooding corals, *Heliopora coerulea* and *Pocillopora damicornis*. Mar Biol. 2002;141: 39–46.

13. Hellberg ME. Footprints on water: the genetic wake of dispersal among reefs. Coral Reefs. 2007;26: 463–473.

14. Eble JA, Rocha LA, Craig MT, Bowen BW. Not all larvae stay close to home: insights into marine population connectivity with a focus on the brown surgeonfish (*Acanthurus nigrofuscus*). J Mar Biol. 2011;2011.

15. Starger CJ, Barber PH, Erdmann MV, Toha AH, Baker AC. Strong genetic structure among coral populations within a conservation priority region, the Bird’s Head Seascape (Papua and West Papua, Indonesia). PeerJ PrePrints; 2013.

16. Dorman JG, Castruccio FS, Curchitser EN, Kleypas JA, Powell TM. Modeled connectivity of *Acropora millepora* populations from reefs of the Spratly Islands and the greater South China Sea. Coral Reefs. 2016;35: 169–179.

17. Sabate F de la S, Sakakura Y, Shiozaki M, Hagiwara A. Onset and development of aggressive behavior in the early life stages of the seven-band grouper *Epinephelus septemfasciatus*. Aquaculture. 2009;290: 97–103.

18. Fisher R, Leis JM. Swimming Speeds in Larval Fishes: From Escaping Predators to the Potential for Long Distance Migration. In: Domenici, P, Kapoor BG, editors. Fish locomotion : an eco-ethological perspective. Enfield, NH: Perspective Science Publishers; 2010. pp. 333–373.
