# Supplementary material for: Larval connectivity patterns of the North Indo-West Pacific coral reefs
Source: PLoS One. 2019 Jul 23;14(7):e0219913. doi: 10.1371/journal.pone.0219913 (PMC6650046; doi:10.1371/journal.pone.0219913)
Supplement: S3 Appendix — (DOCX) [file pone.0219913.s003.docx]

**Table A.** Between-organism difference in connectivity metrics of each reef cell based on the Q-values of the Post-hoc Dunn’s test. All pairwise comparisons were significantly different (Q-values > critical-q value of 2.3877) except for italicized values. Am: *Acropora millepora*, Tg: *Tripneustes gratilla*, E: *Epinephelus* sp.

| Organisms compared | Local Retention | Export Probability | Import Probability | Out-Degree | In-Degree | Mean Distance Exports | Mean Distance Imports |
| --- | --- | --- | --- | --- | --- | --- | --- |
| Am vs Tg | 45.398 | 41.079 | 37.568 | 11.9868 | 52.908 | 51.186 | 50.077 |
| Am vs E | 57.235 | 80.667 | 64.674 | 68.257 | 62.644 | 50.792 | 51.907 |
| Tg vs E | 11.837 | 39.588 | 27.106 | 56.271 | 9.736 | *0.391* | *1.836* |

**Table B.** Between-seasonal matrix difference in connectivity metrics of each reef cell for each model organism based on the Q-values of the Post-hoc Dunn’s test. Most pairwise comparisons were significantly different (Q-values > critical-q value of 2.6310). Italicized values mean no significant difference in pairwise distributions. N.C. means no pairwise comparisons were made. DJF: December, January, February; MAM: March, April, May; JJA: June, July, August; SON: September, October, November.

| Seasonal matrices compared | Local Retention | Export Probability | Import Probability | Out-Degree | In-Degree | Mean Distance Exports | Mean Distance Imports |
| --- | --- | --- | --- | --- | --- | --- | --- |
| ***Acropora millepora*** | | | | | | | |
| DJF vs MAM | 10.4889 | 3.3919 | 3.106 | 1.0958 | 2.6705 | 9.6811 | 6.0193 |
| DJF vs JJA | 8.905 | 3.7653 | 4.8662 | *1.6104* | 3.1025 | 7.0647 | 4.2978 |
| DJF vs SON | 3.8505 | *1.6724* | *1.2564* | 4.0611 | 3.1013 | *0.1066* | *1.2131* |
| MAM vs JJA | *1.5839* | 7.1573 | *1.7602* | *NC* | *0.432* | *2.6131* | 3.085 |
| MAM vs SON | 6.6384 | *1.7195* | *1.8497* | 2.9653 | *0.4308* | 9.5752 | 4.8068 |
| JJA vs SON | 5.0545 | 5.4378 | 3.6098 | *2.4506* | *NC* | 6.9586 | *1.7221* |
| ***Tripneustes gratilla*** | | | | | | | |
| DJF vs MAM | 15.8666 | 5.6425 | 14.1046 | 9.4839 | 10.6051 | 12.5357 | 7.096 |
| DJF vs JJA | 8.1439 | 16.5519 | 10.8661 | 7.8709 | 8.1728 | 8.3855 | *2.5077* |
| DJF vs SON | 3.572 | *0.7597* | *2.0797* | *1.6707* | *1.4748* | *2.4428* | *NC* |
| MAM vs JJA | 7.7227 | 10.9094 | 3.2385 | *1.6129* | *2.4323* | 4.1447 | 4.6089 |
| MAM vs SON | 12.2946 | 4.8828 | 12.0249 | 7.8132 | 9.1302 | 14.9859 | 5.3574 |
| JJA vs SON | 4.5719 | 15.7921 | 8.7864 | 6.2002 | 6.698 | 10.8321 | *0.7686* |
| ***Epinephelus* sp.** | | | | | | | |
| DJF vs MAM | 17.9475 | *1.5904* | 14.0721 | 7.9156 | 9.5216 | 14.9125 | 9.6253 |
| DJF vs JJA | 12.4269 | 15.5257 | 12.1534 | 7.4911 | 7.3112 | 10.4182 | 4.0719 |
| DJF vs SON | 12.4269 | 7.0225 | 13.6409 | 11.7400 | 10.7386 | *1.4081* | *0.7178* |
| MAM vs JJA | 5.5205 | 13.9354 | *1.9187* | *0.4245* | *2.2104* | 4.4993 | 5.5593 |
| MAM vs SON | 6.5782 | 5.4321 | *NC* | 3.8245 | *1.2171* | 13.5055 | 8.9315 |
| JJA vs SON | *1.0577* | 8.5033 | 1.4875 | 4.249 | 3.4275 | 9.0105 | 3.3638 |
