# Supplementary material for: Larval connectivity patterns of the North Indo-West Pacific coral reefs
Source: PLoS One. 2019 Jul 23;14(7):e0219913. doi: 10.1371/journal.pone.0219913 (PMC6650046; doi:10.1371/journal.pone.0219913)
Supplement: S4 Appendix — (DOCX) [file pone.0219913.s004.docx]

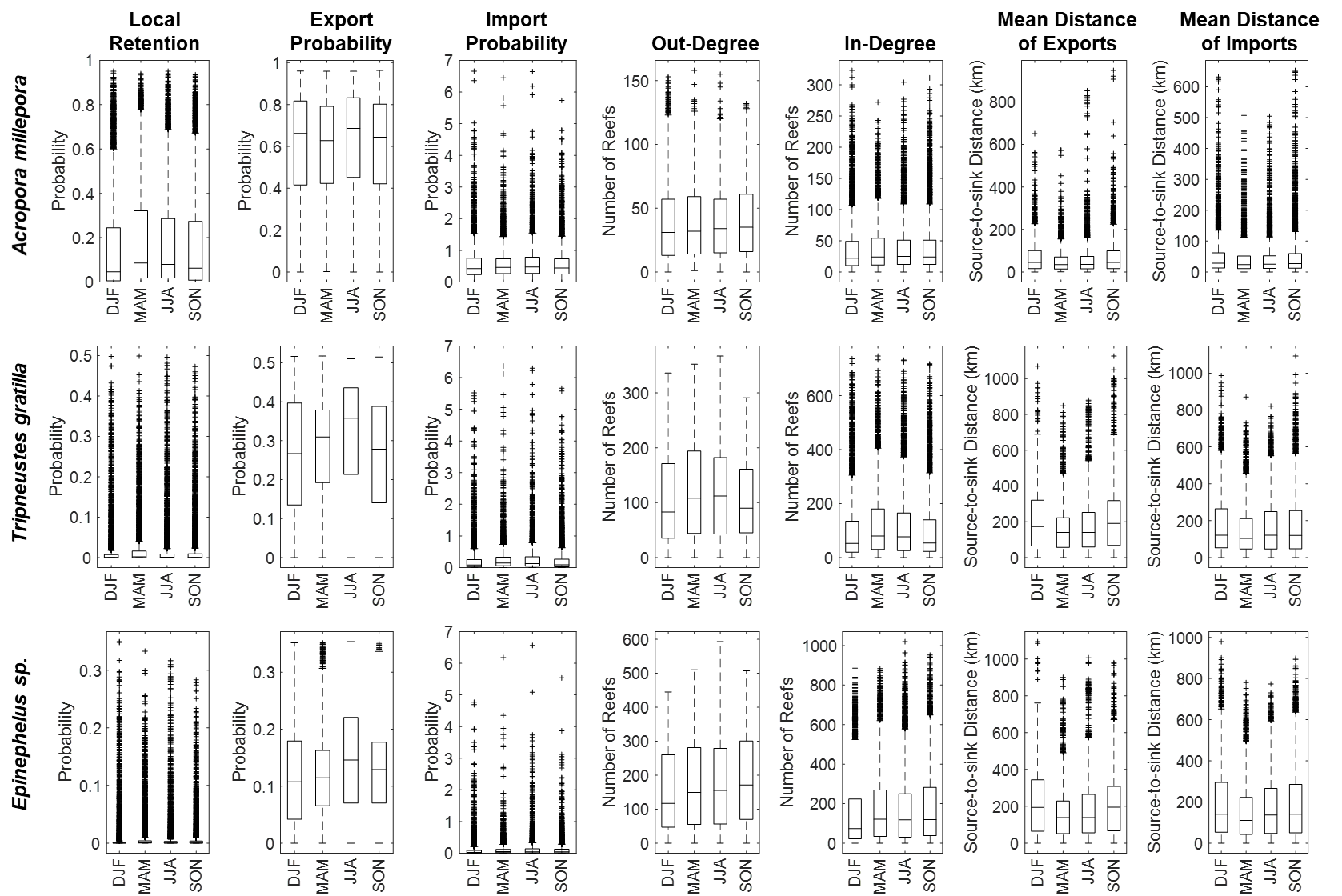


**Figure A.** Between-seasonal matrix comparison of connectivity metrics for each organism. All distributions were significantly different based on Kruskal-Wallis one-way analysis of variance (p < 0.001).


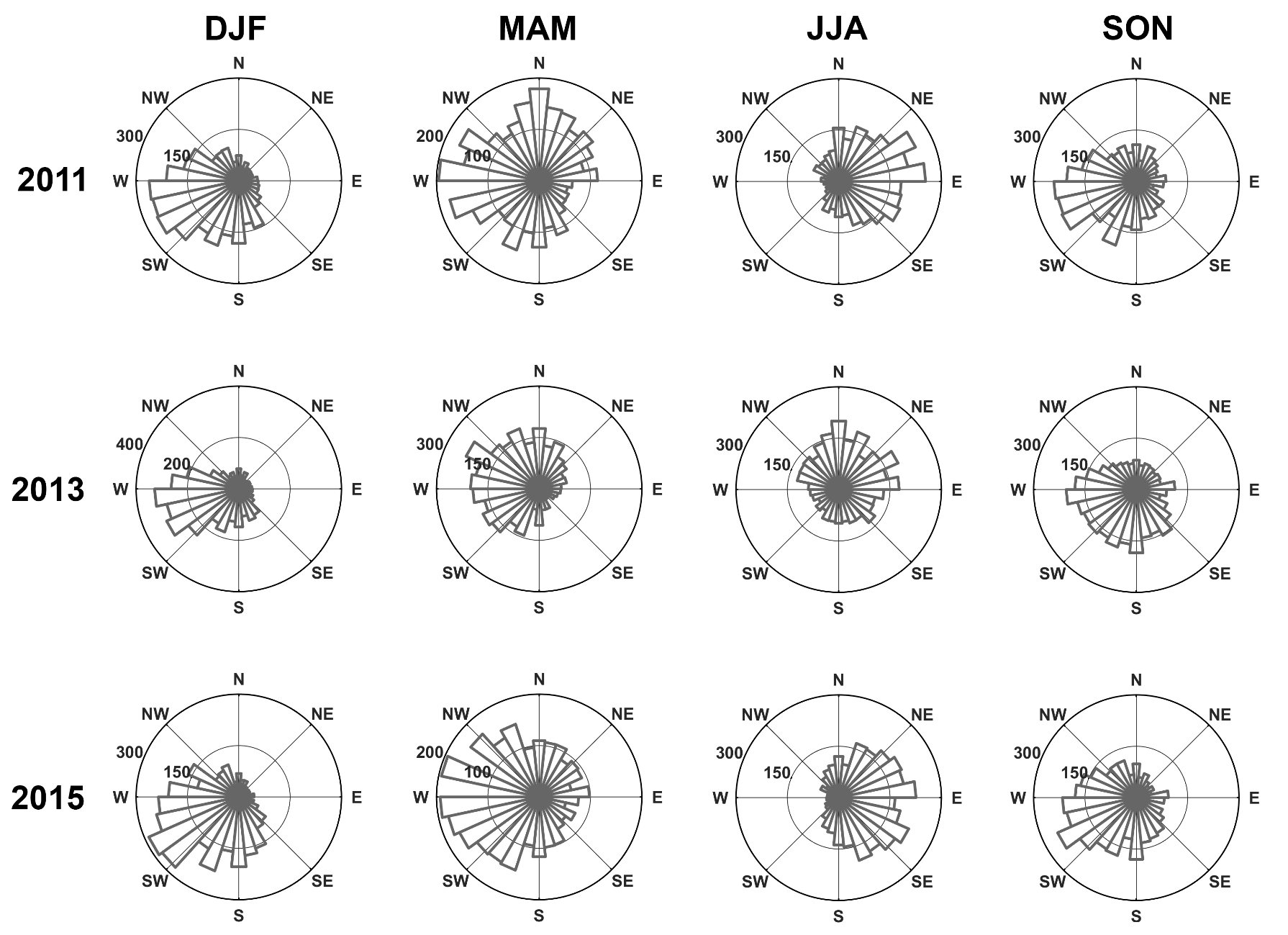


**Figure B.** Temporal comparison of mean direction of exports of each reef cell for *Acropora millepora* matrices. Columns have similar simulation seasons while rows have similar years.


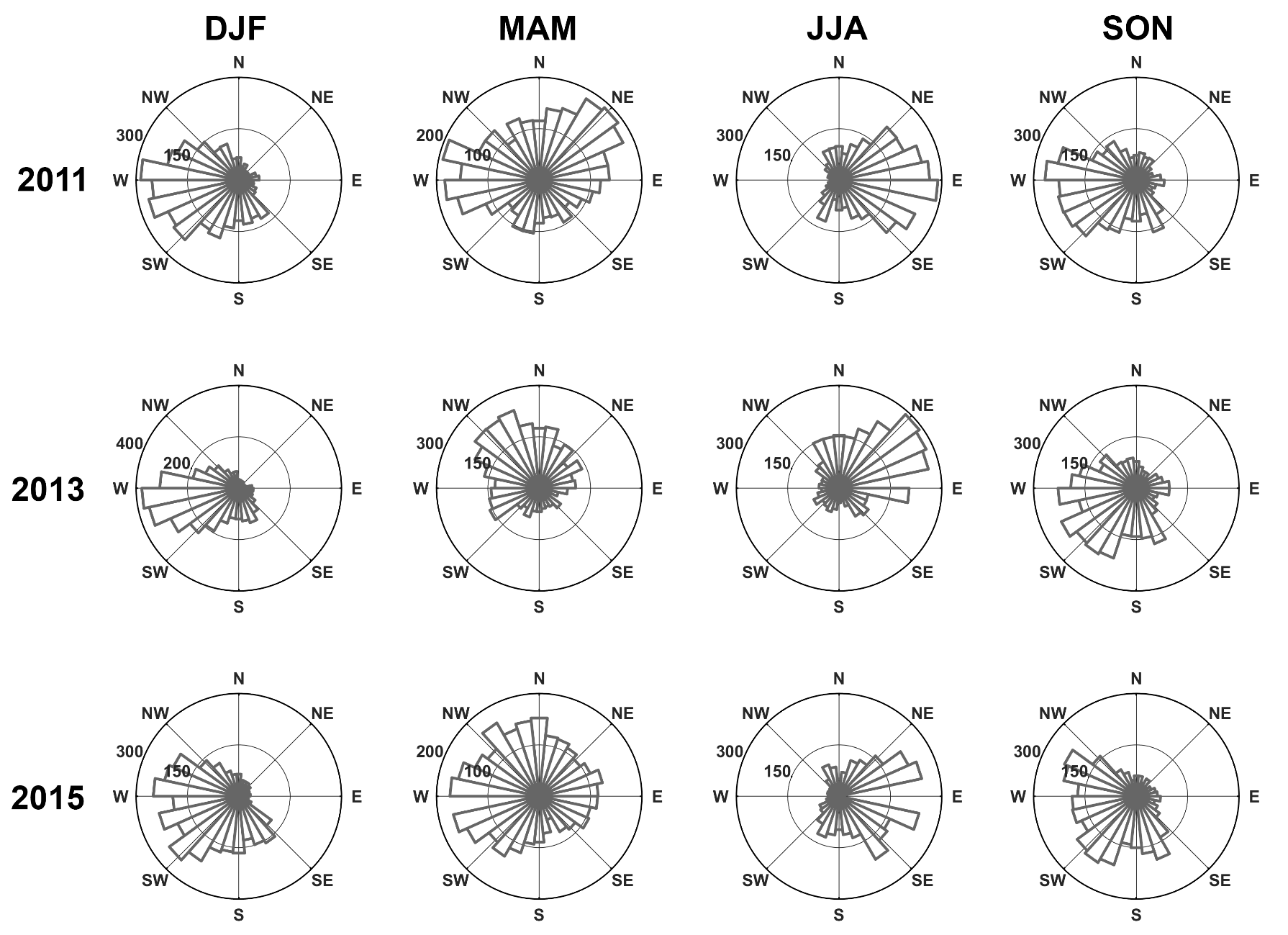


**Figure C.** Temporal comparison of mean direction of exports of each reef cell for *Tripneustes gratilla* matrices. Columns have similar simulation seasons while rows have similar years.


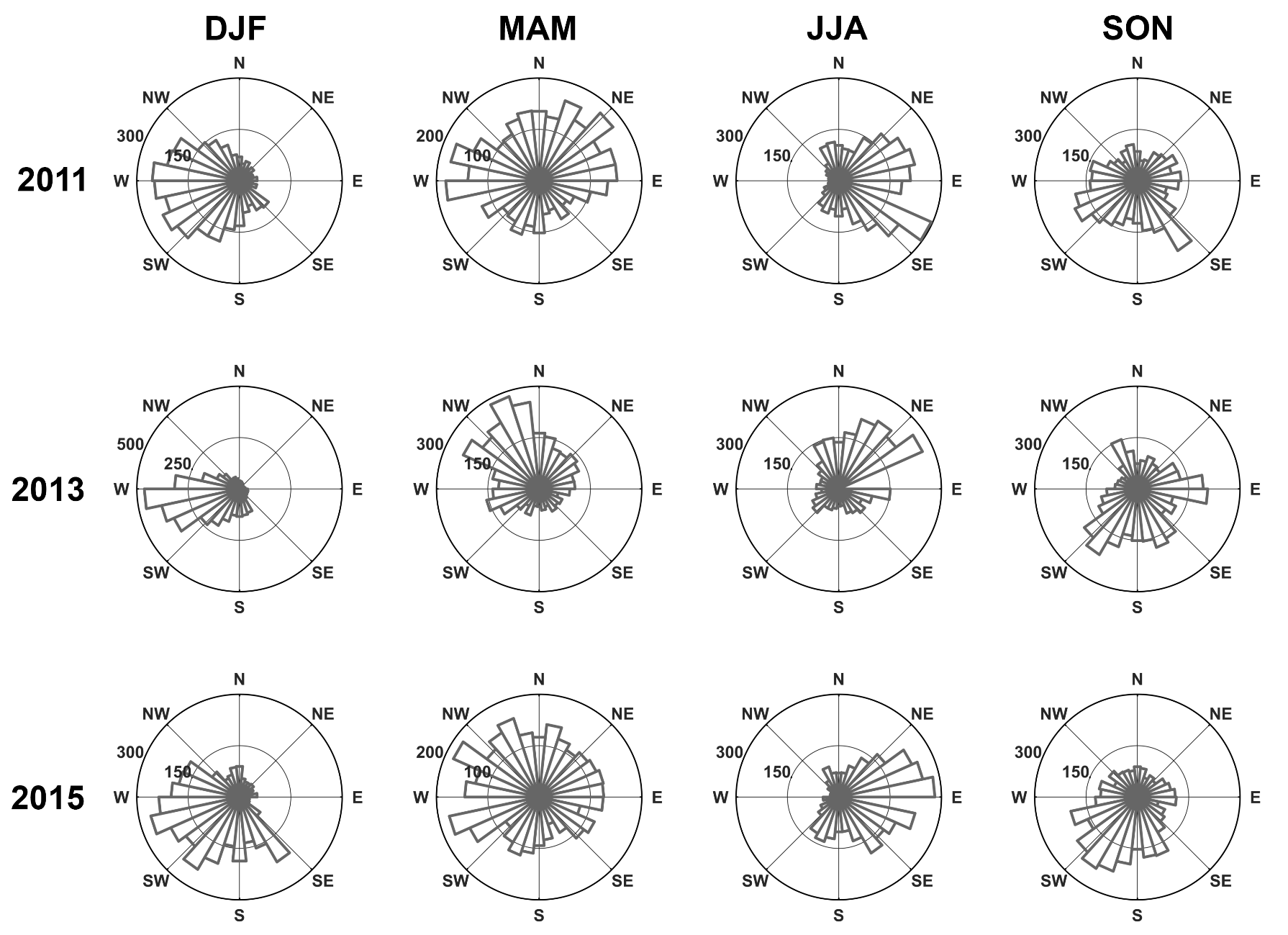


**Figure D.** Temporal comparison of mean direction of exports of each reef cell for *Epinephelus* sp. matrices. Columns have similar simulation seasons while rows have similar years.
