# Supplementary material for: Larval connectivity patterns of the North Indo-West Pacific coral reefs
Source: PLoS One. 2019 Jul 23;14(7):e0219913. doi: 10.1371/journal.pone.0219913 (PMC6650046; doi:10.1371/journal.pone.0219913)
Supplement: S5 Appendix — (DOCX) [file pone.0219913.s005.docx]

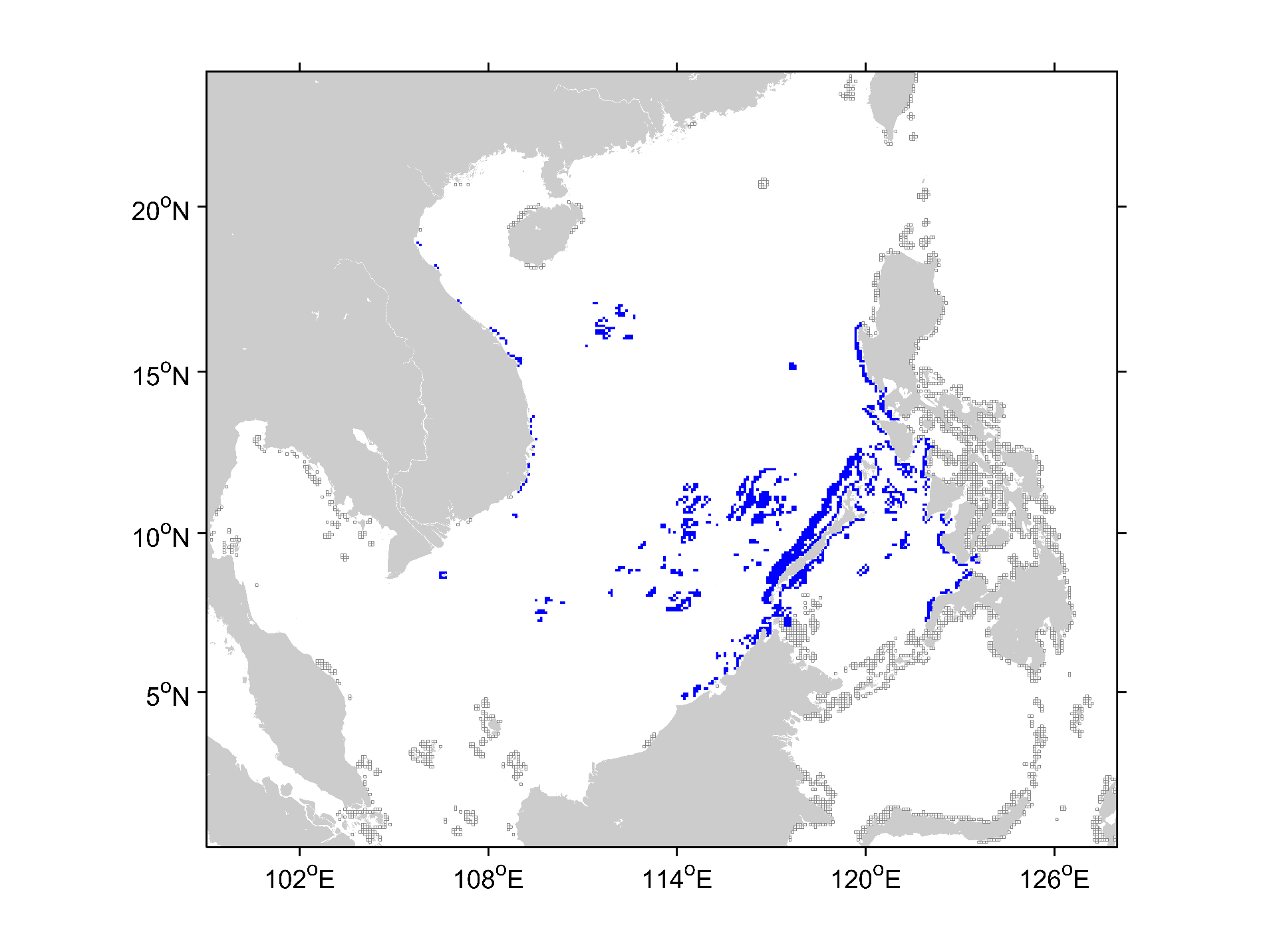


**Figure A.** Clusters of covarying connectivity patterns based on the Bray-Curtis Dissimilary matrix of the source and sink connections of the three model organisms dynamically cut at MCS = 160. Reefs boxed in gray were outliers excluded from the clustering.


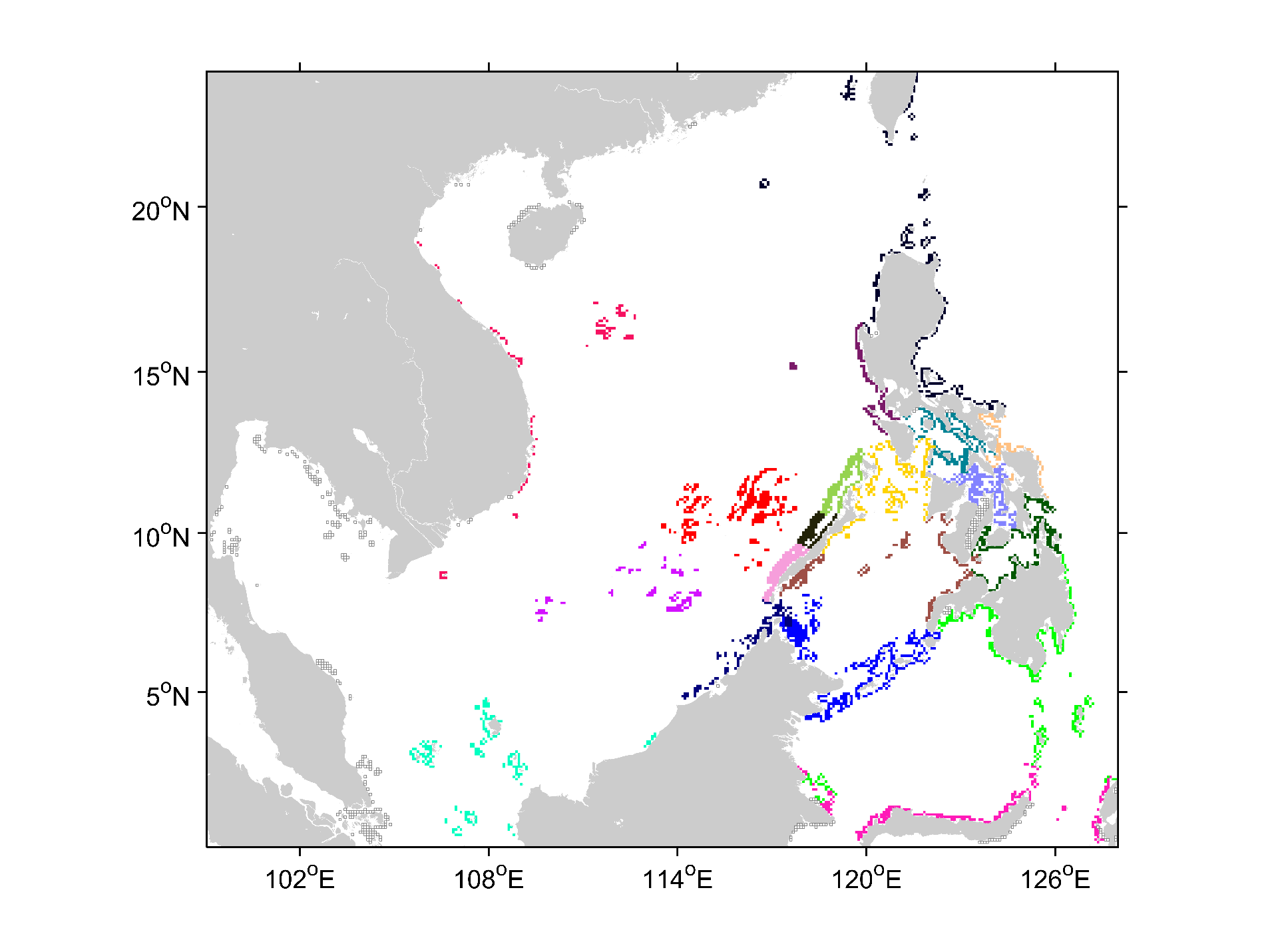


**Figure B.** Clusters of covarying connectivity patterns based on the Bray-Curtis Dissimilary matrix of the source and sink connections of the three model organisms dynamically cut at MCS = 75. Each color represents a unique cluster. Reefs boxed in gray were outliers excluded from the clustering.


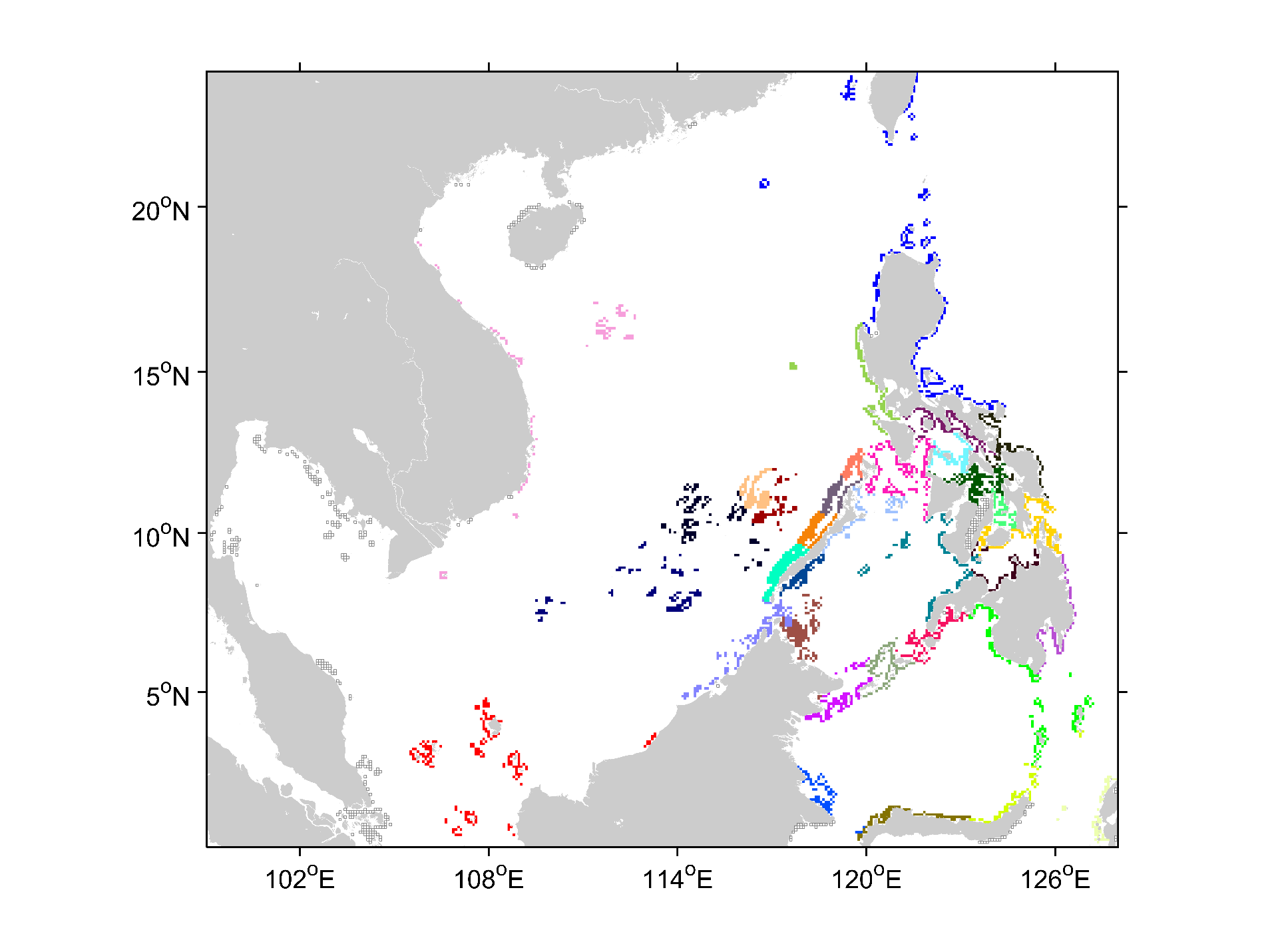


**Figure C.** Clusters of covarying connectivity patterns based on the Bray-Curtis Dissimilary matrix of the source and sink connections of the three model organisms dynamically cut at MCS = 50. Each color represents a unique cluster. Reefs boxed in gray were outliers excluded from the clustering.


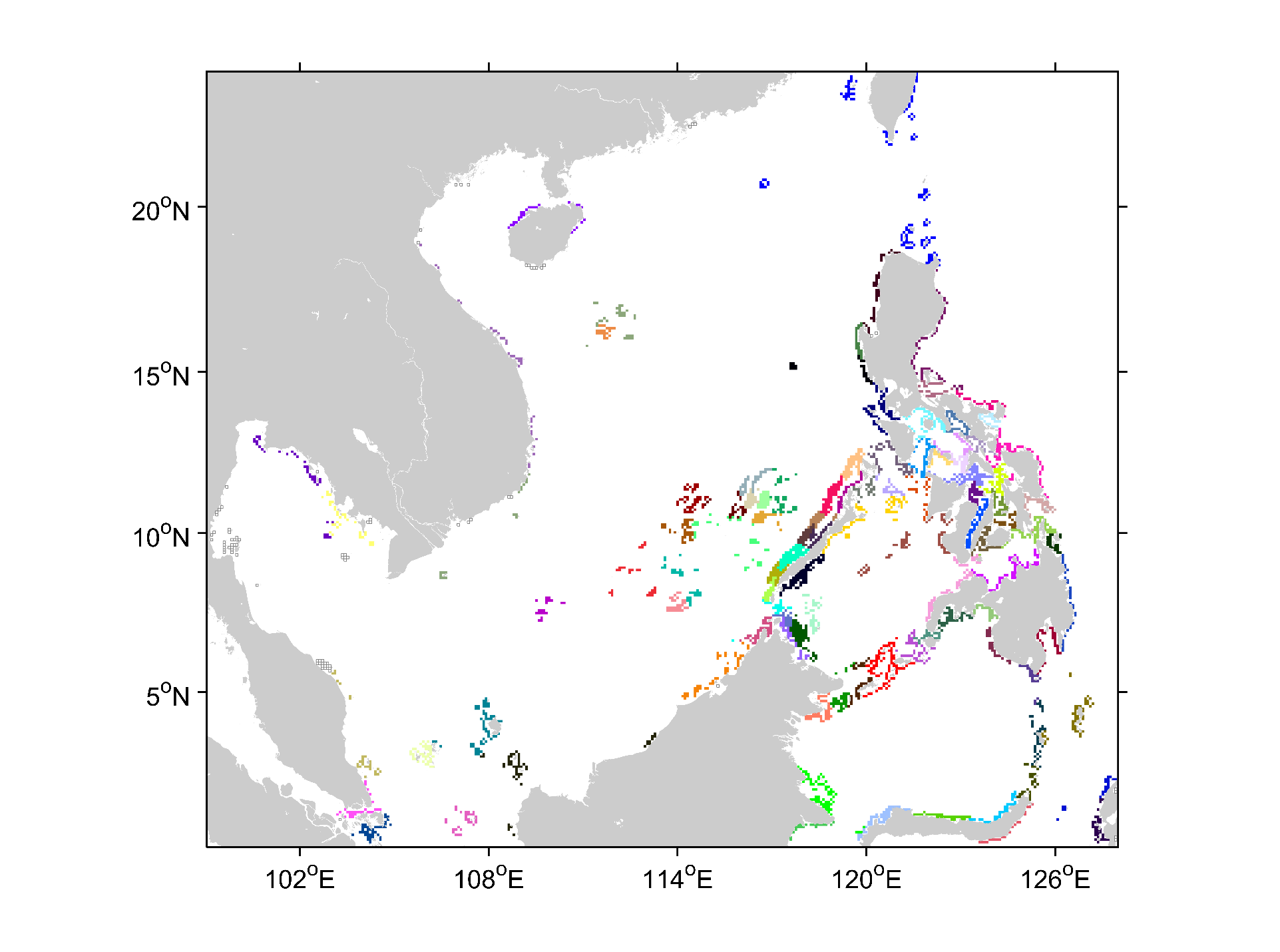


**Figure D.** Clusters of covarying connectivity patterns based on the Bray-Curtis Dissimilary matrix of the source and sink connections of the three model organisms dynamically cut at MCS = 20. Each color represents a unique cluster. Reefs boxed in gray were outliers excluded from the clustering.
